# Structural basis of DSF recognition by its receptor RpfR and its regulatory interaction with the DSF synthase RpfF

**DOI:** 10.1371/journal.pbio.3000123

**Published:** 2019-02-04

**Authors:** Evan J. Waldron, Daniel Snyder, Nicolas L. Fernandez, Emily Sileo, Daigo Inoyama, Joel S. Freundlich, Christopher M. Waters, Vaughn S. Cooper, Matthew B. Neiditch

**Affiliations:** 1 Department of Microbiology, Biochemistry, and Molecular Genetics, New Jersey Medical School, Rutgers, State University of New Jersey, Newark, New Jersey, United States of America; 2 Department of Microbiology and Molecular Genetics, and Center for Evolutionary Biology and Medicine, University of Pittsburgh, Pittsburgh, Pennsylvania, United States of America; 3 Department of Microbiology and Molecular Genetics and the BEACON Center for the Study of Evolution in Action, Michigan State University, East Lansing, Michigan, United States of America; 4 Department of Pharmacology, Physiology, and Neuroscience, New Jersey Medical School, Rutgers, State University of New Jersey, Newark, New Jersey, United States of America; University of North Carolina, UNITED STATES

## Abstract

The diffusible signal factors (DSFs) are a family of quorum-sensing autoinducers (AIs) produced and detected by numerous gram-negative bacteria. The DSF family AIs are fatty acids, differing in their acyl chain length, branching, and substitution but having in common a *cis*-2 double bond that is required for their activity. In both human and plant pathogens, DSFs regulate diverse phenotypes, including virulence factor expression, antibiotic resistance, and biofilm dispersal. Despite their widespread relevance to both human health and agriculture, the molecular basis of DSF recognition by their cellular receptors remained a mystery. Here, we report the first structure–function studies of the DSF receptor regulation of pathogenicity factor R (RpfR). We present the X-ray crystal structure of the RpfR DSF-binding domain in complex with the *Burkholderia* DSF (BDSF), which to our knowledge is the first structure of a DSF receptor in complex with its AI. To begin to understand the mechanistic role of the BDSF–RpfR contacts observed in the biologically important complex, we have also determined the X-ray crystal structure of the RpfR DSF-binding domain in complex with the inactive, saturated isomer of BDSF, dodecanoic acid (C12:0). In addition to these ligand–receptor complex structures, we report the discovery of a previously overlooked RpfR domain and show that it binds to and negatively regulates the DSF synthase regulation of pathogenicity factor F (RpfF). We have named this RpfR region the RpfF interaction (FI) domain, and we have determined its X-ray crystal structure alone and in complex with RpfF. These X-ray crystal structures, together with extensive complementary in vivo and in vitro functional studies, reveal the molecular basis of DSF recognition and the importance of the *cis*-2 double bond to DSF function. Finally, we show that throughout cellular growth, the production of BDSF by RpfF is post-translationally controlled by the RpfR N-terminal FI domain, affecting the cellular concentration of the bacterial second messenger bis-(3′-5′)-cyclic dimeric guanosine monophosphate (c-di-GMP). Thus, in addition to describing the molecular basis for the binding and specificity of a DSF for its receptor, we describe a receptor–synthase interaction regulating bacterial quorum-sensing signaling and second messenger signal transduction.

## Abstract 2

Many Gram-negative bacteria communicate using quorum-sensing autoinducers called diffusible signal factors (DSFs). Structure-function studies in Burkholderia reveal the interaction of a DSF with its receptor as well as the interaction of the receptor with the DSF synthase.

## Author summary

Communication between many species of gram-negative bacteria is mediated by a family of cell–cell signaling autoinducers (AIs) known as the diffusible signal factors (DSFs). DSFs are fatty acids, containing a signature *cis*-2 double bond critical for their activity. The DSFs differ from one another by their acyl chain length, branching, and the presence of additional double bonds. We have carried out the first structure–function studies of DSF binding to a receptor, regulation of pathogenicity factor R (RpfR). We have determined X-ray crystal structures of the RpfR DSF-binding domain in complex with an active DSF, *cis*-2-dodecenoic acid, and in complex with its inactive saturated isomer, dodecanoic acid (C12:0). These X-ray crystal structures, together with extensive complementary in vivo and in vitro functional studies, reveal the molecular basis of DSF recognition and the importance of the *cis*-2 double bond to DSF function. In addition, we have discovered a previously overlooked domain at the RpfR N-terminus, and we show that this domain binds to and negatively regulates the DSF synthase regulation of pathogenicity factor F (RpfF). We present X-ray crystal structures of this domain alone and in complex with RpfF, which—together with in vivo and in vitro functional studies—describe how RpfR binds to RpfF, negatively regulating the synthesis of its own AI.

## Introduction

Quorum sensing is a form of bacterial cell–cell communication that enables populations of bacteria to synchronize their gene expression in order to coordinate group behaviors such as bioluminescence, sporulation, genetic competence, biofilm formation, motility, and virulence factor expression (reviewed in [1,2]). Quorum sensing is mediated by signaling molecules known as autoinducers (AIs) that bacteria secrete and detect. While gram-positive bacteria communicate using peptide AIs (reviewed in [3,4]), gram-negative bacteria use a panoply of small molecule AIs, including the diffusible signal factors (DSFs) (reviewed in [5]), which are the focus of the studies presented here.

**Fig 1:**
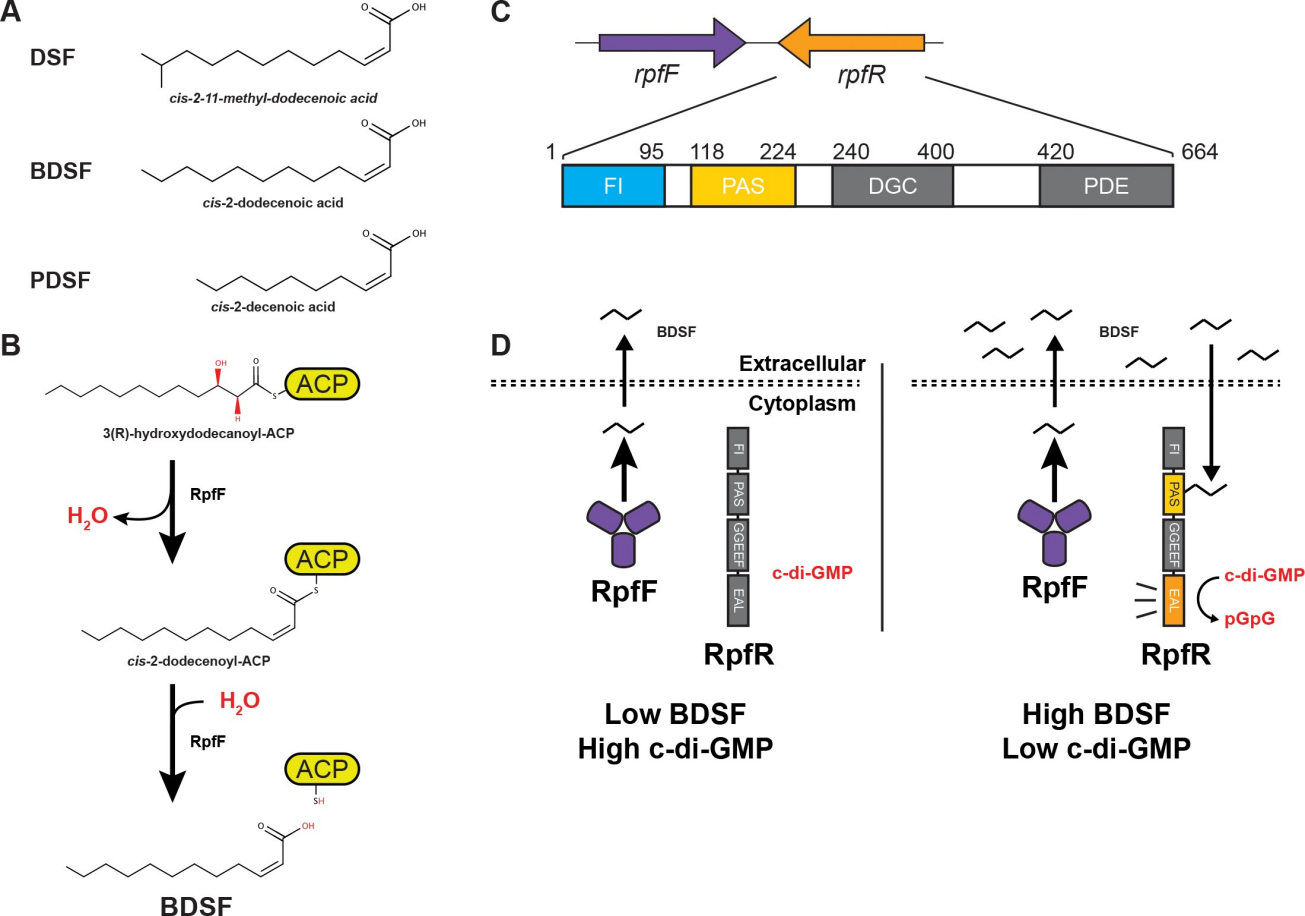
Synthesis and detection of DSF AIs. (A) Structure of DSF (*cis*-2-11-methyl-dodecenoic acid), BDSF (*cis*-2-dodecenoic acid), and PDSF (*cis*-2-decenoic acid). (B) RpfF synthesizes BDSF in a two-step mechanism. RpfF dehydrates 3-hydroxydodecanoyl-ACP to form the *cis*-2-dodecenoyl-ACP. RpfF hydrolyzes the thioester bond linking the acyl-chain to ACP, releasing holo-ACP and free BDSF. (C*)* Schematic representation of RpfR. (Top) The genomic orientation of *rpfF and rpfR* show that they are convergently transcribed. (Bottom) A schematic representation of RpfR_Ct_. The FI domain (residues 1–95) and PAS domain (residues 118–224) are depicted in blue and yellow, respectively. The approximate positions of the DGC and PDE domains (gray) were determined by sequence homology. Linker regions between domains are shown in white. (D) Previously established BDSF-signaling model. (Left panel) At low levels of BDSF, RpfR PDE activity is low and c-di-GMP levels are high. (Right panel) At elevated concentrations, BDSF binds to the RpfR PAS domain, triggering RpfR PDE-mediated hydrolysis of c-di-GMP to pGpG. ACP, acyl carrier protein; AI, autoinducer; BDSF, *Burkholderia* DSF; c-di-GMP, bis-(3′-5′)-cyclic dimeric guanosine monophosphate; DGC, diguanylate cyclase; DSF, diffusible signal factor; FI, RpfF interaction; PAS, Per-Arnt-Sim; PDE, phosphodiesterase; PDSF, *Pseudomonas* DSF; pGpG, 5′-phosphoguanylyl-(3′,5′)-guanosine; RpfF, regulation of pathogenicity factor F; RpfR, regulation of pathogenicity factor R.

DSFs (Fig 1A) are a family of fatty acid AIs produced and detected by a wide range of gram-negative bacteria, including important pathogens [6]. DSFs regulate virulence factor expression, antibiotic resistance, and additional traits important for adaptation within biofilms and biofilm-associated infections [7–11]. While DSFs can differ in their acyl chain length, branching, and substitution, a common chemical feature shared among all DSFs is a *cis*-2 double bond, which is required for signaling activity (reviewed in [12]). The commonality of the *cis*-2 double bond as well as the structural diversity in DSFs is evident when comparing the canonical DSF (*cis*-11-methyl-2-dodecenoic acid), which was discovered in *Xanthomonas campestris* pv. *campestris*, with both the *Burkholderia* DSF (BDSF) (*cis*-2-dodecenoic acid), which was first identified in *Burkholderia cenocepacia*, and the *Pseudomonas* DSF (PDSF) (*cis-*2-decenoic acid), which was discovered in *Pseudomonas aeruginosa* (Fig 1A) [7,13,14].

The enzyme RpfF catalyzes the synthesis of DSF family signals through the dehydration of 3-hydroxyacyl thioesters attached to acyl carrier protein (ACP), producing the *cis*-2 double bond (Fig 1B). Subsequent hydrolysis of the *cis*-2 acyl thioester by RpfF releases the free fatty acid DSF AI from ACP [15,16]. While structural studies have examined DSF synthesis [17–19], the structural basis of DSF–receptor interactions are unexplored.

The DSF receptor RpfR is conserved in numerous gram-negative bacteria, including, among others, *Escherichia coli*, *B*. *cenocepacia*, *Cronobacter turicensis*, *Serratia marcescens*, *Yersinia enterocolitica*, and *Enterobacter cloacae*. RpfR was predicted to contain three domains, namely Per-Arnt-Sim (PAS), diguanylate cyclase (DGC), and phosphodiesterase (PDE) domains (Fig 1C) [20–22]. PAS domains are ligand-binding domains present in all kingdoms of life, and they are capable of detecting a multitude of signals, including small molecules, light, redox, as well as the conformational state of other proteins (reviewed in [23,24]). Therefore, the RpfR PAS domain was predicted to bind DSF [22]. DGC domains, such as that found in RpfR, produce the bacterial second messenger bis-(3′-5′)-cyclic dimeric guanosine monophosphate (c-di-GMP), whose increased concentration is associated with biofilm formation and sessile behavior. PDE domains hydrolyze c-di-GMP to linear 5′-phosphoguanylyl-(3′,5′)-guanosine (pGpG), reducing the cellular concentration of c-di-GMP, which commonly triggers biofilm dispersal and virulence factor expression (reviewed in [25]). BDSF binding to RpfR was previously shown to trigger its PDE activity, decreasing the intracellular concentration of c-di-GMP and favoring biofilm dispersal (Fig 1D) [20,22].

Here, we present a structure–function analysis of RpfR, revealing detailed mechanistic insight into DSF sensing. Additionally, we identified receptor-mediated control of DSF production via a direct interaction of RpfR with the DSF synthase RpfF. The foundation of this work are four X-ray crystal structures. The first two structures presented are those of the RpfR PAS domain in complex with BDSF or the saturated inactive isomer of BDSF, dodecanoic acid (C12:0). Structural comparison of these models along with in vivo functional analysis reveals the molecular basis for BDSF-binding specificity and the role of the DSF *cis*-2 double bond in signal perception. Additionally, we discovered that in contrast to prior predictions [22], RpfR in fact contains an overlooked N-terminal domain that binds to RpfF, inhibiting DSF synthesis. Consistent with its function, we refer to the RpfR N-terminal domain as the RpfF interaction (FI) domain. To explore the mechanism of RpfR-mediated RpfF inhibition, we determined the X-ray crystal structures of the RpfR FI domain alone and in complex with RpfF. These crystal structures, along with extensive in vivo and in vitro studies, reveal the mechanistic basis of this regulation and its contribution to restraining BDSF synthesis and elevating the intracellular concentration of c-di-GMP. Since DSF signaling commonly regulates cellular behaviors, including biofilm formation, biofilm dispersal, swarming motility, extracellular protease production, antibiotic resistance, and other virulence-associated phenotypes in important human and plant pathogens [9,20,26–32], the mechanistic insight into DSF sensing and synthesis presented here set the stage for developing inhibitors important for human health and agriculture.

## Results

### Structure of the RpfR PAS domain bound to dodecanoic acid

**Fig 2:**
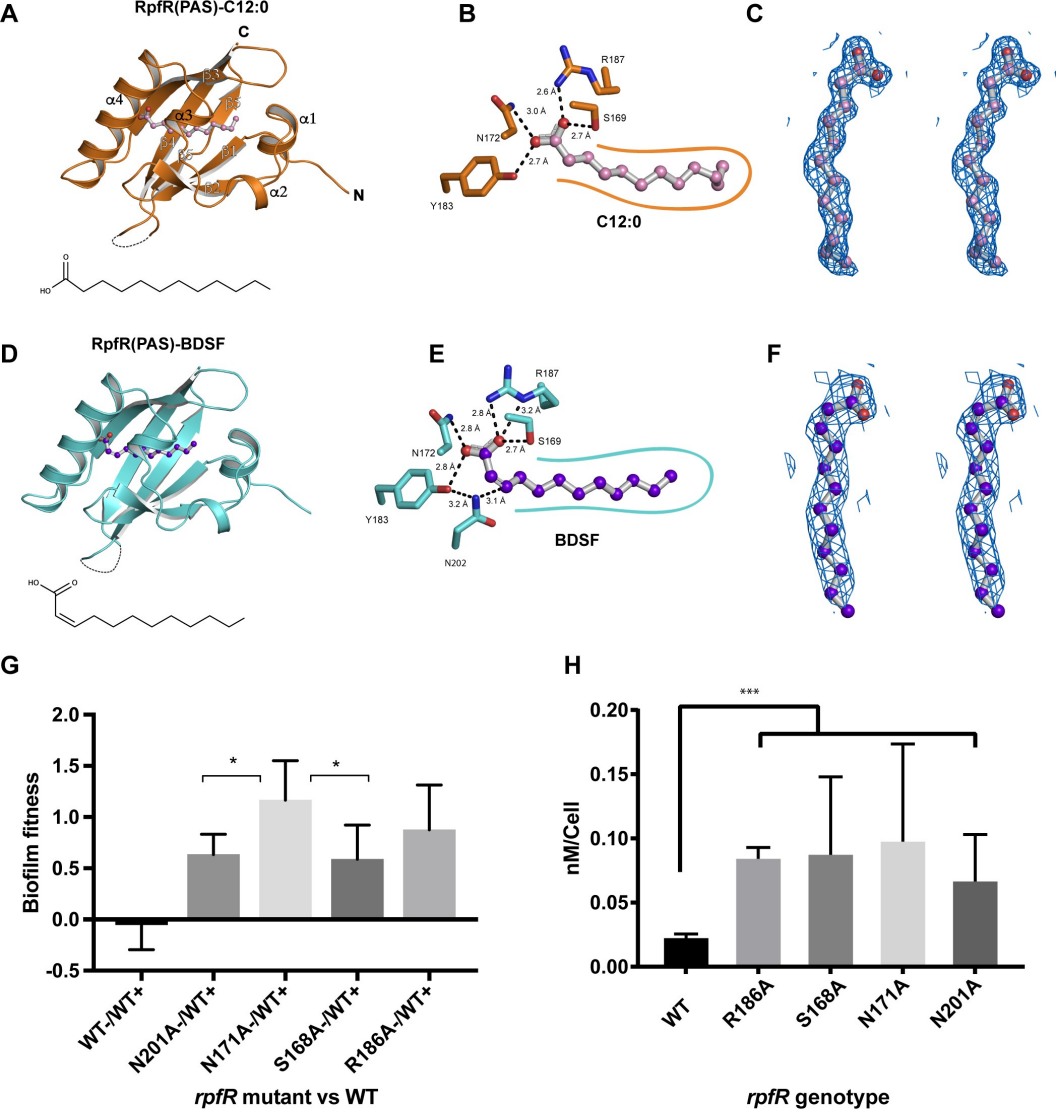
The X-ray crystal structures for RpfR_Ct_(PAS)–C12:0 and RpfR_Ct_(PAS)–BDSF. (A) Cartoon representation of RpfR_Ct_(PAS) (orange) in complex with C12:0 (pink/red balls and gray sticks). Disordered residues 207–212 are shown as a dashed line. C12:0 alone is depicted in the same orientation as the bound ligand below the model. (B) C12:0 (pink/red balls and gray sticks) interacting with the hydrophilic RpfR_ct_(PAS)-binding site residues (orange sticks). Hydrogen bonds (black dashed lines) between hydrophilic residues and the C12:0 carboxylic acid group are shown with their measured distances. RpfR–C12:0 hydrophobic interactions are depicted as a smooth orange contour line. (C) Stereo diagram of C12:0 (pink/red balls and gray sticks) and corresponding 2F_o_-F_c_ electron density contoured at 1.0 σ. (D) Cartoon representation of RpfR_ct_(PAS) (cyan) in complex with BDSF (purple/red balls and gray sticks). Disordered residues 206–212 are shown as a dashed line. Below the structure, BDSF alone is depicted in the same orientation of the modeled ligand. (E) BDSF (purple/red balls and gray sticks) forming hydrogen bonds (black dashed lines with their measured distances) with the same RpfR_ct_(PAS)-binding site residues as C12:0. N202 forms an additional interaction with C3 of BDSF. R187 forms an additional hydrogen bond with the BDSF carboxylic acid. RpfR–BDSF hydrophobic interactions are depicted as a smooth cyan contour line. (F) Stereo diagram of BDSF and corresponding 2F_o_-F_c_ electron density contoured at 1.0 σ (purple/red balls and gray sticks). (G) Biofilm fitness advantages of *B*. *cenocepacia* strains containing chromosomally encoded RpfR_Bc_ BDSF-binding site point mutations in direct competition with WT strain HI2424. Fitness is the difference in selective rate constants (*s*) over 24 h of attachment and biofilm assembly on a polystyrene bead. Each mutant is significantly more fit than WT (ANOVA with Tukey posthoc testing, *P* < 0.001), and N171A is more fit than N201A or S168A (*P* < 0.05) [34,35]. (H) Measurement of the average c-di-GMP pool per cell at 24 h in planktonic cultures of WT *B*. *cenocepacia* or strains containing RpfR_Bc_ BDSF-binding site point mutants. C-di-GMP levels are significantly greater in the mutant strains than in the WT (***Welch’s unpaired *t* test, *P* < 0.0006). The numerical values underlying panels G and H can be found in S1 Data. BDSF, *Burkholderia* DSF; C3, carbon 3; C12:0, dodecanoic acid; c-di-GMP, bis-(3′-5′)-cyclic dimeric guanosine monophosphate; DSF, diffusible signal factor; GMP; PAS, Per-Arnt-Sim; WT, wild-type.

To gain insight into the molecular recognition of BDSF by RpfR, we crystallized and determined the structure of the *C*. *turicensis* RpfR PAS domain (Fig 2A, S1 Table). Without adding fatty acid to the crystallization experiment, we observed prominent electron density in the PAS domain core that was consistent with a 12-carbon fatty acid ligand (Fig 2A–2C). Intriguingly, even when an excess of BDSF was included during crystallization, the electron density was consistent with a fatty acid that did not contain a *cis-2* double bond (Fig 2C). These data suggested that RpfR_Ct_(PAS) copurified with a tightly bound fatty acid likely originating from the *E*. *coli* overexpression host. Indeed, using liquid chromatography–mass spectrometry (LC-MS), we determined that the ligand is the saturated isomer of BDSF, C12:0 (S1 Fig), which *E*. *coli* is known to produce endogenously [33]. Thus, we built and refined the 1.5 Å resolution X-ray crystal structure of RpfR_Ct_(PAS)–C12:0 (Fig 2A–2C).

While C12:0 is an inactive isomer of BDSF, the RpfR_Ct_(PAS)–C12:0 structure provided the first insight into how fatty acids interact with RpfR and, as shown below, proved to be important for comparative analysis. In the RpfR_Ct_(PAS)–C12:0 structure, the C12:0 carboxylic acid group forms multiple hydrogen bonds with a set of conserved hydrophilic residues (S169, N172, Y183, and R187) at one end of the ligand-binding pocket (Fig 2B). At the other end of the pocket, the C12:0 hydrophobic acyl tail is surrounded by conserved hydrophobic residues (Fig 2A and 2B, S2A Fig). While the RpfR_Ct_(PAS)–C12:0 structure provided insight into RpfR fatty acid binding, a mechanistic understanding of how RpfR recognizes BDSF demanded determination of the bona fide RpfR_Ct_(PAS)–BDSF structure.

### Purification and X-ray crystal structure of the RpfR PAS domain–BDSF complex

BDSF was previously demonstrated to trigger RpfR_Ct_ phosphodiesterase activity [20]. To obtain RpfR_Ct_(PAS) bound to the functionally active ligand BDSF, rather than the inactive ligand C12:0, we overexpressed and purified the RpfR_Ct_(PAS) domain from *E*. *coli* engineered to express BDSF. It is notable that while *E*. *coli* contains a highly conserved *rpfR* homolog named the gene modulating RNase II (*gmr*), it lacks a DSF synthase and, to our knowledge, does not produce a DSF AI [21,34]. We speculate, however, that *E*. *coli* Gmr PDE activity could be regulated by C12:0. Regardless, *E*. *coli* was previously demonstrated to synthesize BDSF when transformed with an expression plasmid containing *rpfF*_Bc_ [15,30]. Therefore, we purified RpfR_Ct_(PAS) from *E*. *coli* coexpressing RpfF_Bc_. The purified RpfR_Ct_(PAS) was then denatured and LC-MS used to identify the released ligand (S3 Fig). Indeed, the bound ligand was BDSF, confirming that we had purified the RpfR_Ct_(PAS)–BDSF complex.

The RpfR_Ct_(PAS)–BDSF complex was crystallized and its structure determined to a resolution of 2.3 Å (Fig 2D–2F, S1 Table). Comparison of the RpfR_Ct_(PAS)–BDSF and RpfR_Ct_(PAS)–C12:0 structures reveals that the ligands adopt different conformations, resulting from the absence or presence of the *cis*-2 double bond in C12:0 or BDSF, respectively. Furthermore, while RpfR_Ct_ arginine (Arg) 187 makes a single H-bond to C12:0, it adopts a different rotameric configuration in the presence of BDSF, forming an additional H-bond between its δ nitrogen and the BDSF carboxylic acid (Fig 2B and 2E).

Based on the measured distances and atom types (Fig 2B and 2E), the position of BDSF in the RpfR_Ct_(PAS)-binding pocket appears to be influenced by an interaction between BDSF and the conserved residue asparagine (Asn) 202 (Fig 2E, S2A Fig and S4 Fig). Specifically, we propose that the Asn202 side chain amide nitrogen interacts with the electron deficient C_β_ (C3) of BDSF. Below, we explore the physiological importance of the RpfR_Ct_(PAS)–BDSF interactions observed in the crystal structure.

### Functional studies of BDSF binding to RpfR

Relative to wild-type *B*. *cenocepacia*, strains with RpfR proteins containing mutations predicted to disrupt the binding of BDSF to RpfR_Bc_(PAS) should be less sensitive to BDSF, exhibit lower PDE activity, display elevated levels of c-di-GMP, and have increased competitive fitness in biofilms due to elevated biofilm production. We engineered mutations RpfR_Bc_–S168A,–N171A,–R186A, and–N201A (corresponding to the above-described and conserved RpfR_Ct_ BDSF-interacting residues S169, N172, R187, and N202 [S2A Fig]) in the chromosomal copy of *rpfR* and competed them 1:1 with wild-type under a daily cycle of biofilm formation, dispersal, and reattachment [10,35]. Consistent with the interactions observed in the RpfR_Ct_(PAS)–BDSF crystal structure, each mutation produced large and significant fitness advantages, with selective coefficients (*s*) ranging from 0.58 to 1.17 (Fig 2G). Furthermore, each of these mutations resulted in a significant increase in cellular c-di-GMP relative to the wild-type strain (Fig 2H). While it is possible that the site-directed mutations affected RpfR stability and, in turn, the ability of RpfR to respond to BDSF, we found that purified full-length wild-type RpfR_Bc_, RpfR_Bc_–S168A,–N171A,–R186A, and–N201A were comparably soluble (S5 Fig). Finally, in line with the fact that the RpfR_Bc_(PAS) BDSF-binding mutants displayed increased competitive fitness in the in vitro biofilm life cycle assay and elevated levels of c-di-GMP, we note that RpfR_Bc_(PAS) is subject to positive selection under conditions favoring biofilm growth, such as in a cystic fibrosis patient for which increased biofilm production proves advantageous [11].

### Identification and crystallization of a previously undescribed RpfR N-terminal domain

**Fig 3:**
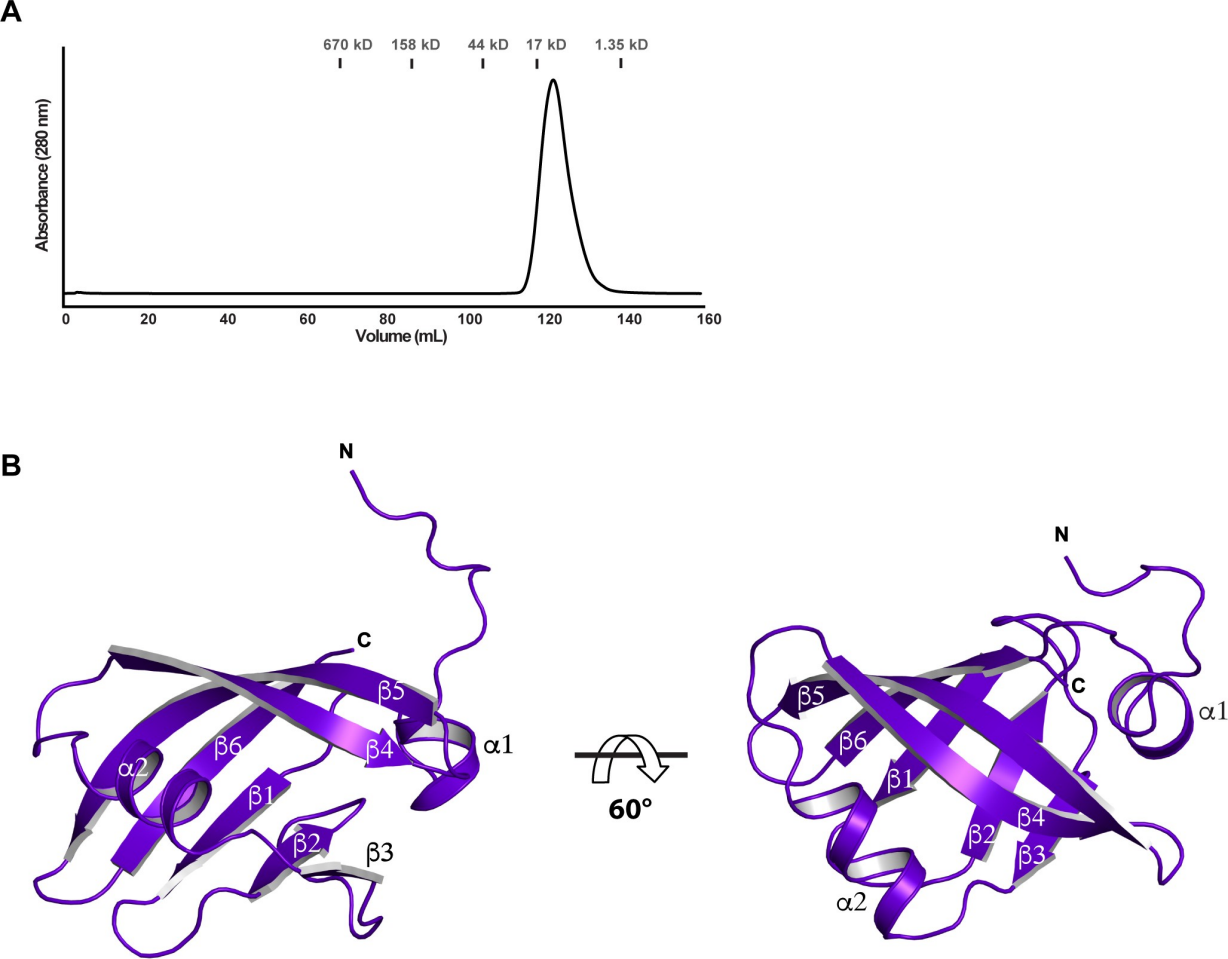
Structure of RpfR_Ct_(FI). (A) A size exclusion chromatogram of RpfR_Ct_(FI). (B) Two views of the RpfR_Ct_(FI) domain are displayed rotated 60° from each other along the horizontal axis. Individual secondary structure elements are labeled. FI, RpfF interaction; RpfF, regulation of pathogenicity factor F; RpfR, regulation of pathogenicity factor R.

Previous studies identified three RpfR domains: PAS, DGC (GGDEF), and PDE (EAL) [20–22] (Fig 1C). We observed that the undescribed region of RpfR (residues 1–117) was highly conserved among all RpfR homologues (S2B Fig). This led us to hypothesize the presence of a new domain located N-terminal to the BDSF-binding PAS domain. Indeed, we found that a construct containing the first 95 residues of RpfR_Ct_ (RpfR_Ct_[1–95]) was soluble and well folded, as determined by size exclusion chromatography (Fig 3A). As described below, RpfR_Ct_(1–95) was amenable to crystallographic analysis.

### Structure of the RpfR N-terminal domain

We determined the X-ray crystal structure of the RpfR_Ct_(1–95) to a resolution of 1.2 Å using the single-wavelength anomalous dispersion (SAD) method (Fig 3B, S1 Table). RpfR_Ct_(1–95) adopts a Profilin-like fold consisting of an N-terminal α-helix and a central six-stranded antiparallel β-sheet with a single α-helix connecting β3 to β4 (Fig 3B, S2B Fig and S6A Fig) [36,37]. Distance-matrix alignment (DALI) and PDBeFold searches of the Protein Data Bank (PDB) show the closest RpfR_Ct_(1–95) structural homologues to be PAS, Ca^2+^channels-chemotaxis receptors (Cache), and cyclic GMP-specific phosphodiesterase-adenylyl cyclase-FhlA (GAF) domains; however, canonical PAS domains contain five-stranded β-sheets (S6B Fig) [23], and Cache domains are periplasmic domain proteins that contain an additional N-terminal α-helix that extends through the membrane [38]. Like RpfR_Ct_(1–95), GAF domains contain six-stranded antiparallel β-sheets [23,39]; however, unlike GAF domain proteins, RpfR_Ct_(1–95) does not possess a pair of interacting α-helices below its β-sheet (S6C Fig).

Consistent with the fact that RpfR_Ct_(1–95) is neither a PAS, Cache, or GAF domain, protein BLAST (BLASTP) searches using the RpfR_Ct_(1–95) and RpfR_Bc_(1–94) amino acid sequences return neither PAS, Cache, nor GAF domain proteins [40]. Based on the above structural and sequence analysis, we conclude that while the RpfR N-terminal domain is structurally similar to PAS, Cache, and GAF domains, it is a new domain with a Profilin-like fold. As detailed below, this RpfR domain binds to and negatively regulates RpfF; thus, we refer to it as the RpfF interaction (FI) domain.

### RpfR(FI) interacts with RpfF

**Fig 4:**
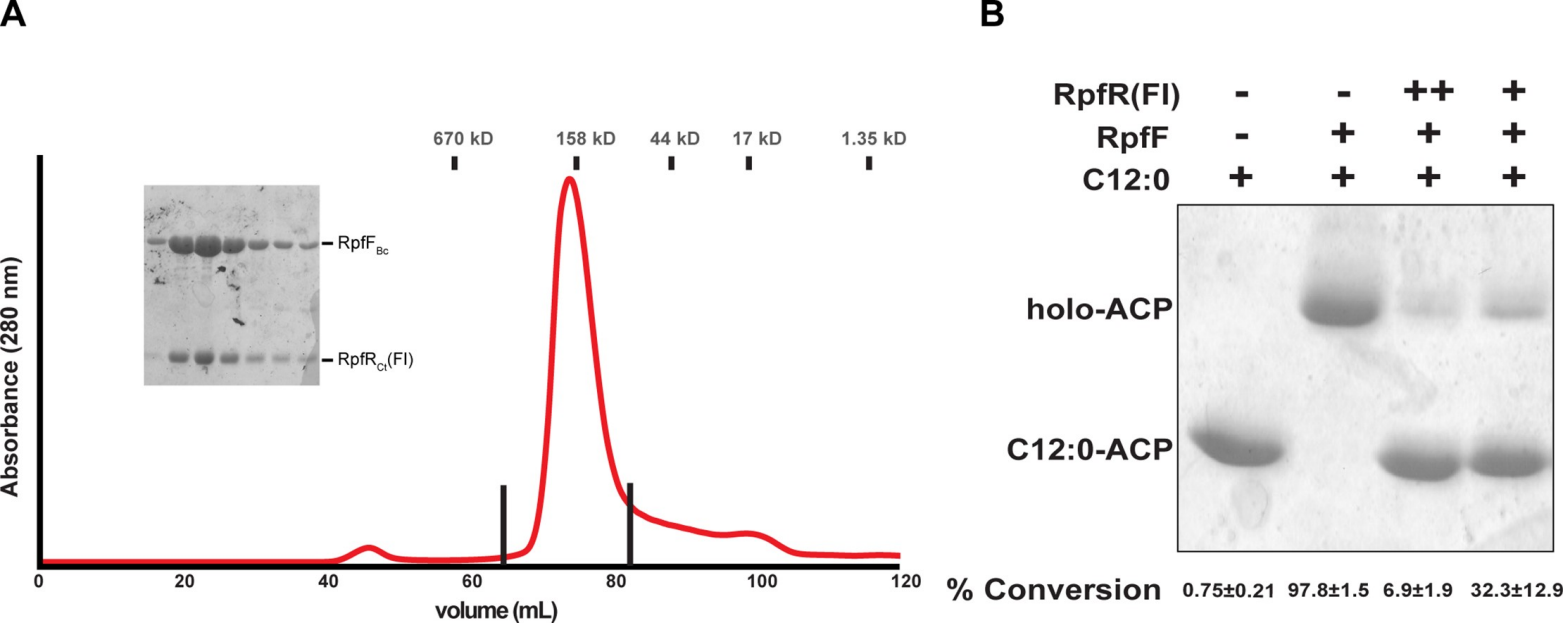
RpfR(FI) binds to RpfF, regulating its thioesterase activity. (A) Size exclusion chromatogram of RpfF_Bc_–RpfR_Ct_(FI). Fractions between the vertical lines were analyzed by 15% SDS-PAGE (inset). The positions of molecular weight standards are indicated above the trace. (B) Conformation sensitive gel electrophoresis of RpfF_Bc_ thioesterase activity in the presence and absence of RpfR_Ct_(FI). The substrate was C12:0–ACP. Average percent conversion of substrate and standard deviation from three separate experiments is shown beneath each lane. The gel shown is a representative example of the three experiments. The numerical values underlying panel B can be found in S1 Data. ACP, acyl carrier protein; C12:0, dodecanoic acid; FI, RpfF, interaction; RpfF, regulation of pathogenicity factor F; RpfR, regulation of pathogenicity factor R.

Initial evidence suggesting that RpfR(FI) and RpfF interact came from studies in which an RpfR_Bc_ construct containing both the FI and PAS domains (RpfR_Bc_[FI–PAS]) and RpfF_Bc_ were coexpressed in *E*. *coli* to obtain RpfR_Bc_(FI–PAS) bound to BDSF. As discussed above, RpfF_Bc_ was employed in these experiments because it was previously shown to produce BDSF when overexpressed in *E*. *coli* [15,30]. During our experiments to obtain BDSF-bound RpfR_Bc_(FI–PAS), RpfR_Bc_(FI–PAS) containing an N-terminal hexahistidine (His_6_) affinity tag copurified with nontagged RpfF_Bc_ (S7 Fig). As this interaction was not observed when His_6_-tagged RpfR_Ct_(PAS) was purified following coexpression with RpfF_Bc_, we hypothesized that RpfR_Bc/Ct_(FI) binds to RpfF_Bc_. To test this hypothesis, we coexpressed RpfF_Bc_ and RpfR_Ct_(FI) and purified the RpfF_Bc_–RpfR_Ct_(FI) complex (Fig 4A).

### RpfR(FI) inhibits RpfF thioesterase activity

Based on the fact that RpfR(FI) and RpfF form a complex, we hypothesized that RpfR(FI) regulates RpfF enzymatic activity. To test this hypothesis, we generated *E*. *coli* 4′-phosphopantetheinyl ACP (holo-ACP_Ec_) and charged it with C12:0. C12:0-charged ACP_Ec_ (C12:0–ACP_Ec_) is a substrate for the analysis of in vitro RpfF thioesterase activity [15,17], which we then measured in either the absence or presence of RpfR_Bc_(FI) (Fig 4B). In the absence of RpfR_Bc_(FI), C12:0–ACP_Ec_ was readily converted by RpfF_Bc_ to holo-ACP_Ec_. In the presence of RpfR_Bc_(FI), conversion of C12:0–ACP_Ec_ substrate by RpfF_Bc_ was significantly reduced (Fig 4B). These results indicate that RpfR(FI)_Bc_ inhibits RpfF_Bc_ thioesterase activity.

### RpfF-RpfR(FI) complex structure

**Fig 5:**
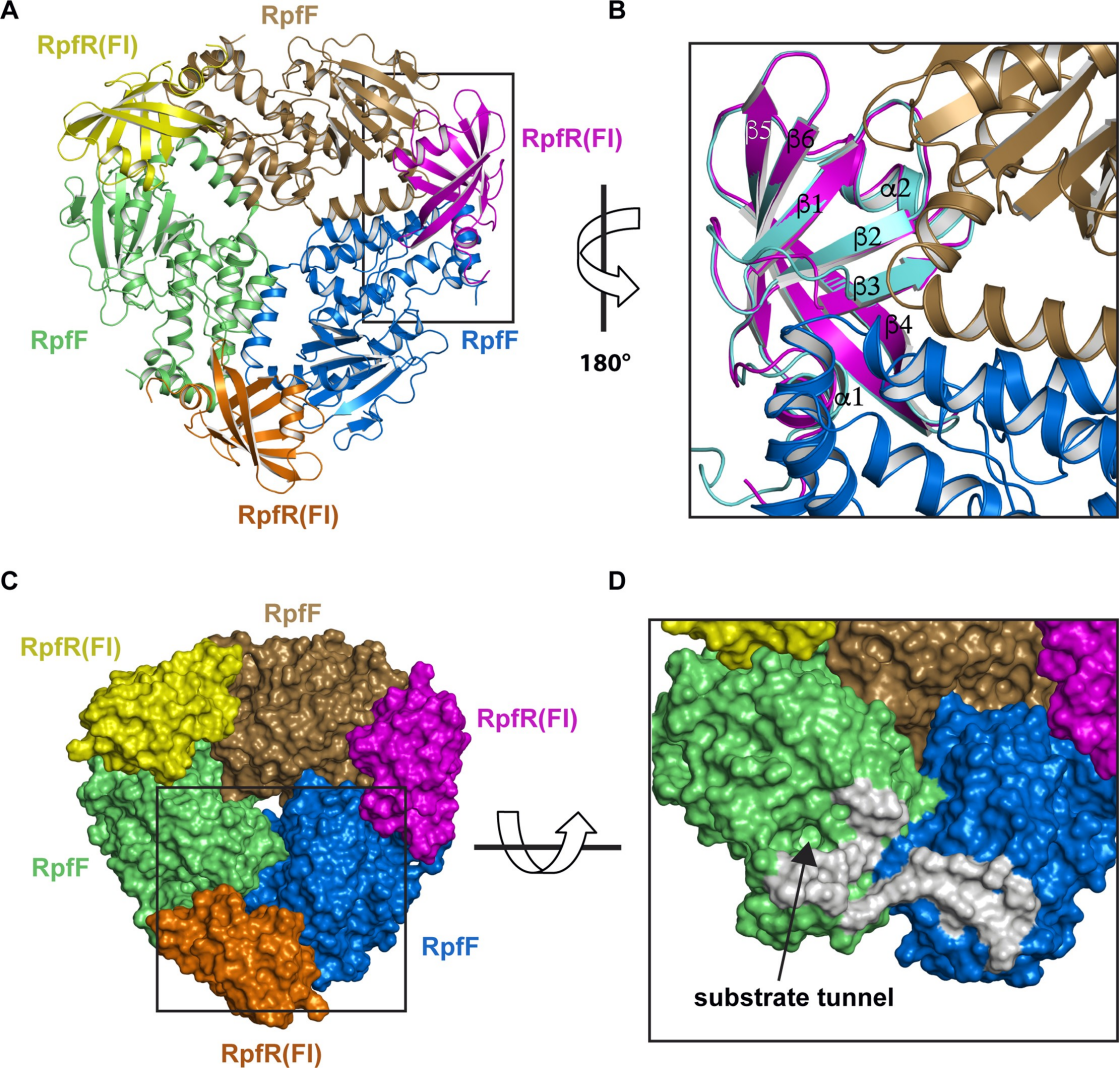
Structure of the RpfF_Bc_–RpfR_Ct_(FI) complex. (A) Cartoon representation of the RpfF_Bc_–RpfR_Ct_(FI) heterohexamer. RpfF_Bc_ monomers are colored lime, sand, and marine. RpfR_Ct_(FI) monomers are colored yellow, magenta, and orange. (B) Expanded (and rotated 180°) view of the area enclosed by the rectangle in A with the structure of RpfR_Ct_(FI) alone (cyan) aligned with RpfR_Ct_(FI) in complex with RpfF_Bc_ (magenta). (C) Surface representation of the hexamer (colored as in A). (D) Expanded and tilted view of the area enclosed by the rectangle in C orientated looking into the substrate tunnel entrance (for clarity RpfR_Ct_[FI] is omitted). RpfF_Bc_ residues that interact with RpfR_Ct_(FI) are depicted as a gray surface. FI, RpfF interaction; RpfF, regulation of pathogenicity factor F; RpfR, regulation of pathogenicity factor R.

To understand how RpfR(FI) inhibits RpfF thioesterase activity, we determined the RpfF_Bc_–RpfR_Ct_(FI) X-ray crystal structure to a resolution 2.0 Å (Fig 5, S1 Table). RpfF_Bc_–RpfR_Ct_(FI) is a heterohexamer consisting of three RpfR_Ct_(FI) and RpfF_Bc_ protomers. The heterohexamer is generated by 3-fold crystallographic symmetry of the asymmetric unit containing RpfF_Bc_–RpfR_Ct_(FI). Each RpfR_Ct_(FI) protomer simultaneously interacts with two RpfF_Bc_ protomers near their homodimerization interfaces (Fig 5A–5C). This interaction places RpfR(FI)_Ct_ in close proximity to the RpfF_Bc_ substrate tunnel entrance (Fig 5C and 5D). It is also worth noting that the RpfF_Bc_–RpfR_Ct_(FI) interfacial residues are conserved (S2B and S8 Figs), and this interaction is likely common in bacteria encoding both proteins.

When comparing the structure of RpfF_Bc_ alone (PDB:5FUS) [17] with the structure of RpfF_Bc_–RpfR_Ct_(FI), perhaps the most surprising observation is that RpfF_Bc_–RpfR_Ct_(FI) contains no bound fatty acid (S9A Fig). In the previously determined crystal structure of RpfF_Bc_, C12:0 originating from the *E*. *coli* expression system was identified as a ligand in the RpfF_Bc_ substrate tunnel [17]. Attempts to remove C12:0 prior to biochemical and structural studies were unsuccessful, suggesting that C12:0 may be tightly bound [17]. Consistent with the absence of bound fatty acid in RpfF_Bc_–RpfR_Ct_(FI), structural alignment of RpfF_Bc_–RpfR_Ct_(FI) with RpfF_Bc_ shows that in the complex, phenylalanine (Phe) residues 44 and 88 have adopted a conformation in which they would sterically clash with bound fatty acid if it were present (S9B Fig).

Finally, superposing the structures of RpfR_Ct_(FI) alone with the structure of RpfR_Ct_(FI) in complex with RpfF_Bc_ (Fig 5B) reveals that RpfR_Ct_(FI) undergoes minor conformational changes primarily at the RpfF_Bc_ binding surface. The most significant of these changes occur in RpfR_Ct_(FI) in which the N-terminus of strand β3 extends by one residue, the β2–β3 loop shifts to form contacts with two molecules of RpfF_Bc_ near their homodimerization interface, and the β4–β5 loop becomes buried in the RpfF_Bc_ interface (Fig 5B). Based on the proximity of RpfR(FI)_Ct_ to the RpfF_Bc_ substrate tunnel and its ability to inhibit RpfF thioesterase activity, we propose that RpfR_Ct_(FI) functions to sterically block acyl-ACP substrates from entering the RpfF active site.

### RpfR regulates BDSF and c-di-GMP production in vivo

**Fig 6:**
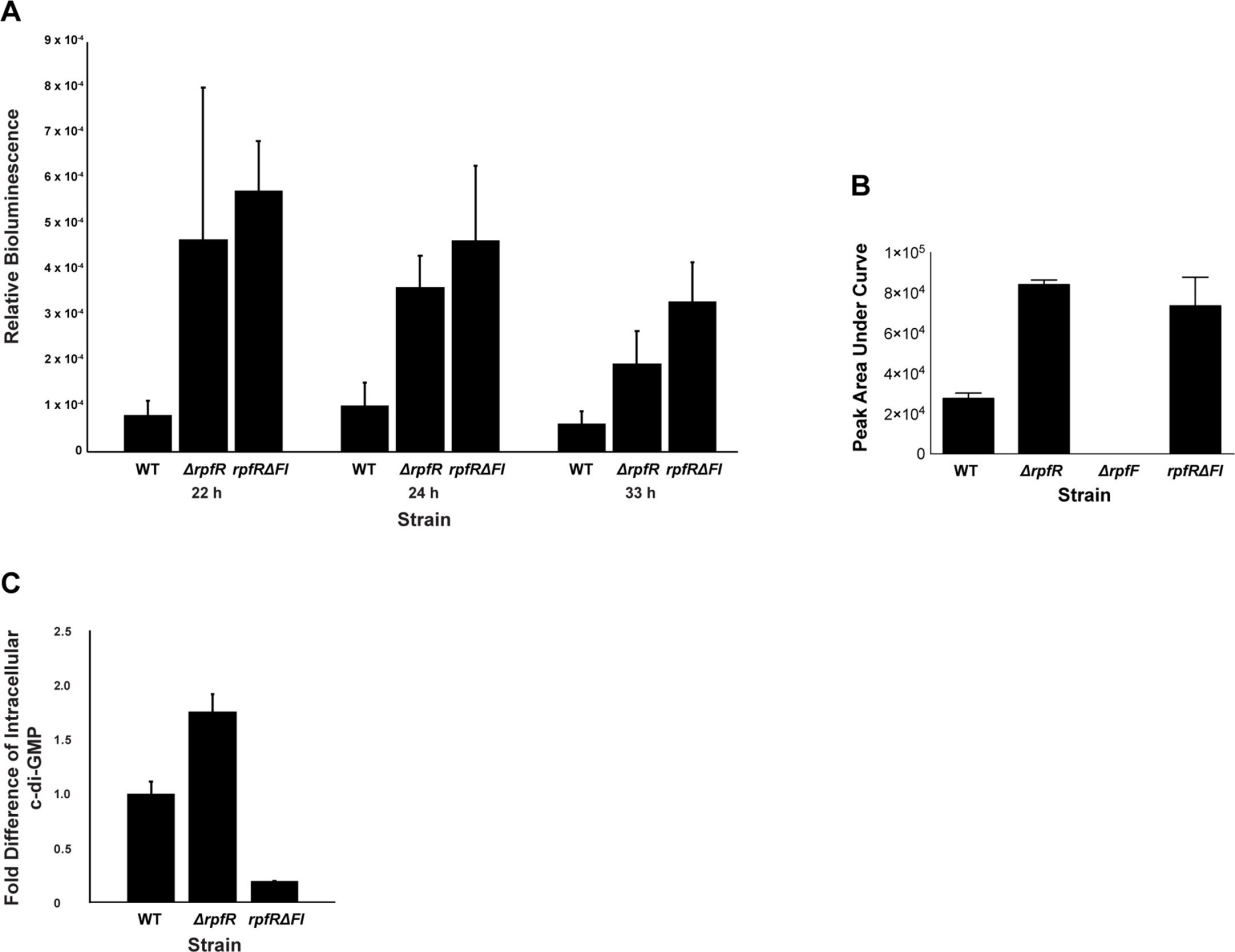
The RpfR FI domain affects the extracellular accumulation of BDSF and the intracellular level of c-di-GMP. (A) The amount of BDSF present in the cell-free supernatant of WT, **Δ***rpfR*, and *rpfR***Δ**FI truncation *B*. *cenocepacia* HI2424 strains was measured using the BDSF bioassay. Relative bioluminescence values are shown for times 22 h, 24 h, and 33 h. (B) The amount of BDSF present in the cell-free supernatant of WT, **Δ***rpfR*, **Δ***rpfF*, and *rpfR***Δ**FI truncation *B*. *cenocepacia* HI2424 strains was measured using mass spectroscopy. *n* = 4, and error bars are 95% confidence intervals. *Indicates a *P* value < .05 determined by one-way ANOVA with Tukey posthoc testing. (C) The fold difference in intracellular c-di-GMP levels in biofilm cells of *B*. *cenocepacia* HI2424 strains **Δ***rpfR* and *rpfR***Δ**FI truncation are shown relative to the levels of the WT strain. The numerical values underlying panels A–C can be found in S1 Data. BDSF, *Burkholderia* DSF; c-di-GMP, bis-(3′-5′)-cyclic dimeric guanosine monophosphate; DSF, diffusible signal factor; FI, RpfF interaction; RpfF, regulation of pathogenicity factor F; RpfR, regulation of pathogenicity factor R; WT, wild-type.

To measure the regulatory effect that RpfR has on RpfF BDSF synthesis in vivo, we employed a *B*. *cenocepacia* BDSF bioassay and mass spectroscopy to monitor the extracellular accumulation of BDSF in wild-type and mutant strains. More specifically, we compared the extracellular concentration of BDSF in cultures of *B*. *cenocepacia* strains that were either wild-type (strain HI2424), had the entire *rpfR* gene deleted, or contained an in-frame deletion of the *rpfR* FI domain (Fig 6A and 6B). The *rpfR* and *rpfR* FI domain deletion strains had significantly increased extracellular levels of BDSF compared to the wild-type strain. Consistent with these in vivo BDSF bioassay results and the above biochemical data, the *B*. *cenocepacia rpfR* deletion strain produced elevated levels of c-di-GMP relative to the wild-type strain under biofilm growth conditions, likely resulting from the deletion of the RpfR PDE domain (Fig 6C). Moreover, the RpfR FI domain deletion strain displayed significantly reduced levels of c-di-GMP, consistent with the elevated levels of BDSF stimulating RpfR PDE activity.

## Discussion

### The role of the BDSF *cis*-2 double bond

The defining feature of the DSF family of AIs that distinguishes them from other fatty acids is their *cis*-2 double bond (Fig 1A) [12]. The *cis*-2 double bond dramatically affects DSF receptor–binding affinity and biological activity. BDSF was shown to tightly bind RpfR_Bc_ (K_d_ = 877 nM), while C12:0 (K_d_ = 800 μM) and *trans*-2 dodecenoic acid (K_d_ = 150 μM) were found to weakly interact with the receptor [22]. Consistent with these results, BDSF was active in a *B*. *cenocepacia* BDSF bioassay, while C12:0 and *trans*-2 dodecenoic acid were inactive [41]. Despite great interest in DSF-signaling systems, the mechanism of molecular recognition employed by DSF receptors such as RpfR to distinguish DSF AIs from other cellular fatty acids, including the *trans*-2 and saturated isomers of DSF AIs, was unknown. As detailed below, we propose that the BDSF *cis*-2 double bond establishes RpfR_Ct_-binding specificity and affinity by enabling unique ligand–receptor contacts and, in contrast to more flexible fatty acids such as C12:0, by paying a lower entropic cost upon receptor binding.

Structural comparison of RpfR_Ct_(PAS)–C12:0 and RpfR_Ct_(PAS)–BDSF reveals mostly subtle conformational changes localized to the RpfR_Ct_(PAS) domain ligand-binding side chains (Fig 2B and 2E). One conformational difference is RpfR_Ct_–R187, which makes an additional H-bond to the BDSF carboxylic acid than it makes to the C12:0 carboxylic acid (Fig 2B and 2E). Interestingly, RpfR_Ct_–R187 can mediate this additional H-bond because it adopts a different rotameric configuration in the BDSF-bound structure. Much of the RpfR_Ct_–R187 side chain is solvent exposed, and we speculate that it could play a role in regulating the activity of the receptor C-terminal enzymatic PDE domain upon BDSF binding.

Based on the measured distances and atom types (Fig 2B and 2E, S4 Fig), BDSF appears to be interacting with the conserved RpfR_Ct_ amino acid Asn202. As an α,β-unsaturated carboxylic acid, BDSF contains an electron deficient C_β_ (C3). We propose that the more electron rich N202 amide nitrogen interacts with the electron deficient C_β_ of BDSF. This interaction cannot occur for the fully saturated C12:0 and would be significantly weaker for *trans*-2 dodecenonic acid because its C_β_ would be suboptimally positioned further away from N202. Additionally, in some RpfR proteins, there is a tyrosine corresponding to RpfR_Ct_–Y183, whose hydroxyl interacts with the N202 side chain amide N-H (Fig 2E). This interaction could further increase the electron density on the N202 amide nitrogen, strengthening its interaction with C_β_ (C3) of BDSF.

Finally, it is important to note that due to the presence of the *cis*-2 or *trans*-2 double bond in BDSF and *trans*-2 dodecenoic acid, respectively, they are more rigid than their saturated isomer C12:0. Theoretically, there is less entropy lost upon receptor binding to BDSF or *trans*-2 dodecenoic acid than upon binding to C12:0. Thus, we speculate that in addition to the specific receptor contacts mediated by the BDSF *cis*-2 double bond, it could contribute to receptor affinity and specificity by lowering the entropic penalty paid in comparison to saturated fatty acids.

### BDSF-mediated quorum sensing is regulated by an interaction between its receptor and synthase

We have discovered a previously unidentified PAS-like domain at the RpfR N-terminus and demonstrated that it binds directly to RpfF, inhibiting BDSF synthesis in vitro and in vivo (Fig 4B; Fig 6A and 6B), ultimately affecting the cellular concentration of c-di-GMP (Fig 6C). Ongoing studies in our labs are examining the molecular basis of this regulation. Due to the proximity of the RpfR FI domain to the RpfF substrate tunnel entrance (Fig 5C and 5D), in all likelihood, RpfR sterically blocks acyl-ACP binding to RpfF and acyl-ACP substrate access to the RpfF active site.

While we have shown that RpfR(FI) interacts with RpfF—tuning or restraining the amount of BDSF produced and secreted—we believe that RpfR(FI) has also evolved to limit the amount of general acyl-ACP substrate cleavage. It was previously demonstrated that RpfF cleaves a broad range of acyl-ACP substrates, producing free fatty acids, which is energetically wasteful, as many of these would not be DSF AI compounds or serve any other known physiological role [15,17,42, 43]. Many bacteria that express RpfF also contain an enzyme, regulation of pathogenicity factor B (RpfB), that salvages free fatty acids by ligating them to coenzyme A (CoA), generating substrates for beta oxidation [43]. RpfB was shown to be important for counteracting RpfF substrate promiscuity, as *rpfB* mutants displayed RpfF-dependent growth defects [43]. It is important to note that bacteria encoding RpfR do not contain RpfB, and how these bacteria might compensate for RpfF substrate cleavage promiscuity was unknown. We propose that an important role for RpfR(FI) is to compensate for the lack of RpfB in these bacteria by tightly regulating RpfF, governing its cleavage of acyl-ACP substrates.

**Fig 7:**
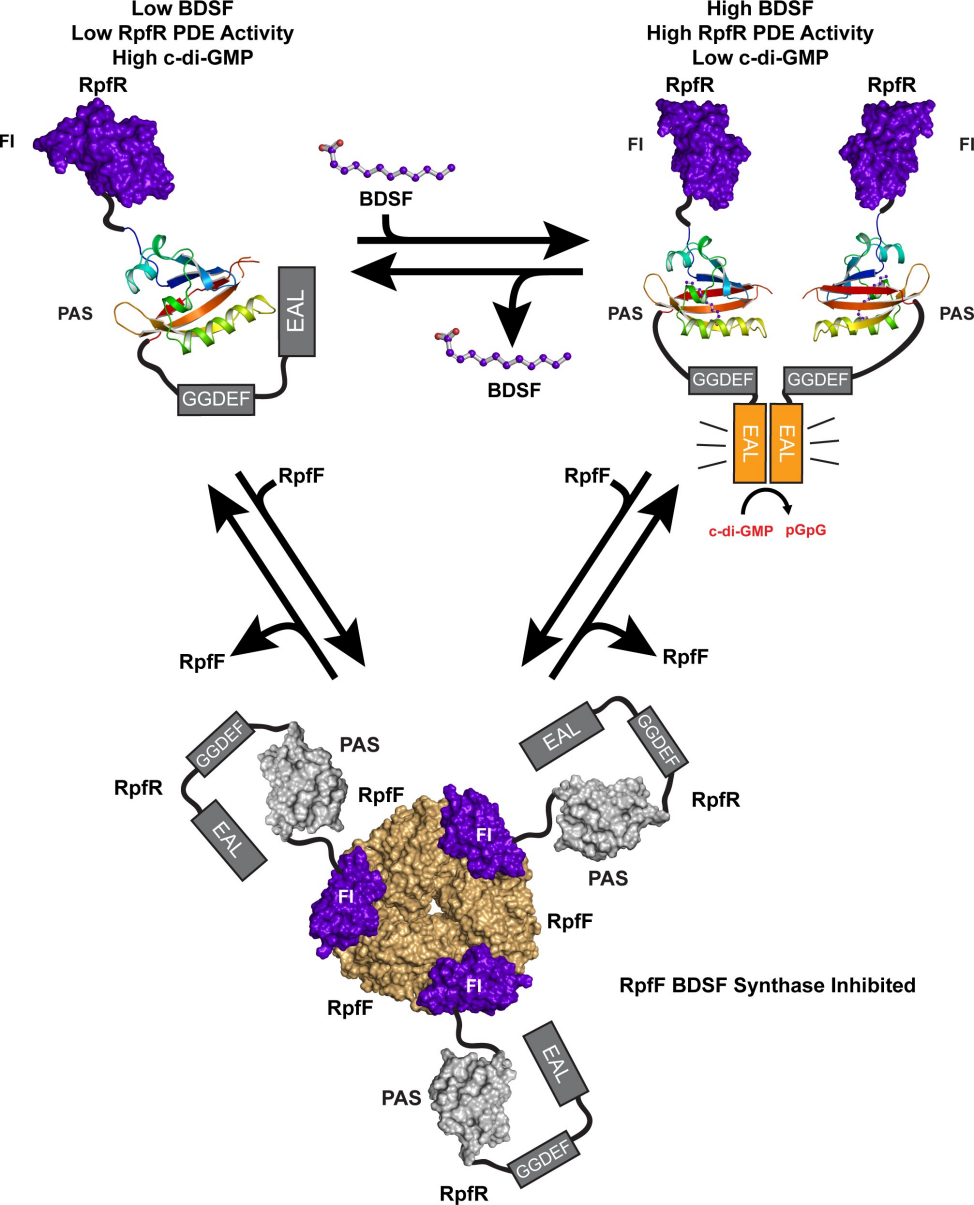
Proposed mechanism of RpfR activation by BDSF and RpfF inhibition by RpfR. At low-cell density (top left), BDSF levels are low and the RpfR PDE domain is minimally active, permitting elevated levels of c-di-GMP and favoring biofilm formation. Here, we depict a theoretical regulatory interaction between the PAS (rainbow cartoon) and PDE (gray box) domains. At high-cell density (top right), BDSF has accumulated and bound to the RpfR PAS domain. This activates the RpfR PDE domain to degrade c-di-GMP to pGpG, favoring biofilm dispersal. Here, we depict RpfR PDE domain dimerization occurring upon BDSF binding; however, PDE activation may also occur via the rearrangement of catalytically inactive (autoinhibited) RpfR dimers. Throughout cellular growth, the RpfR FI domain (purple surface) associates with RpfF (light orange) (bottom), restraining BDSF synthesis, in turn modulating the cellular level of c-di-GMP. BDSF, *Burkholderia* DSF; c-di-GMP, bis-(3′-5′)-cyclic dimeric guanosine monophosphate; DSF, diffusible signal factor; FI, RpfF interaction; PAS, Per-Arnt-Sim; PDE, phosphodiesterase; pGpG, 5′-phosphoguanylyl-(3′,5′)-guanosine; RpfF, regulation of pathogenicity factor F; RpfR, regulation of pathogenicity factor R.

Based on the existing data, we propose the following model for RpfR function (Fig 7). At low-cell density and below a critical concentration of BDSF, the RpfR c-di-GMP PDE has minimal activity [22], and biofilms can form because c-di-GMP levels are elevated. Why the RpfR PDE domain is minimally active in the absence of BDSF is unknown, but we hypothesize that the RpfR PAS domain may directly interact with the PDE domain inhibiting its activity. Throughout cellular growth, RpfR directly regulates RpfF processing of acyl-ACP substrates. More specifically, the newly discovered RpfR FI domain binds RpfF at its acyl-ACP–binding site, limiting its promiscuous cleavage of acyl-ACP substrates. Ultimately, the BDSF concentration reaches a critical concentration in which its binding to the RpfR PAS domain activates RpfR c-di-GMP PDE activity. This activation converts c-di-GMP to pGpG, triggering biofilm dispersal. It is important to note that PDE domains are typically allosterically activated upon dimerization [44–47]. We propose that BDSF binding to the RpfR PAS domain activates RpfR PDE activity by triggering RpfR dimerization or the conformational rearrangement of catalytically inactive (autoinhibited) RpfR dimers. In fact, a similar model was proposed for the regulator of biofilm dispersal of *Pseudomonas aeruginosa* (RbdA) [48]. Finally, what role, if any, BDSF binding to RpfR(PAS) plays in controlling the RpfR(FI)–RpfF association is unknown; however, if BDSF binding to RpfR(PAS) regulates RpfR(FI)–RpfF association, in turn controlling RpfF activity, then BDSF, RpfR, and RpfF comprise a feedback loop regulating BDSF synthesis. Determining whether such a feedback loop exists and elucidating the structural basis of its function is necessary if we are to understand the regulatory controls modulating BDSF synthesis and cell–cell communication.

Finally, we note that PAS and PAS-like domains structurally similar to the RpfR FI domain are common components of histidine kinases as well as other bacterial signal transduction proteins [23]. It will be interesting to determine whether some of these domains, like the FI domain, function to directly regulate the activity of a target enzyme. We propose that these interactions, like the physiologically important interactions identified here between RpfR and the AI BDSF as well as between RpfR and the BDSF synthase RpfF, could be targeted for the development of signaling agonists or antagonists and could serve as therapeutics modulating critical bacterial developmental processes including biofilm formation, biofilm dispersal, and virulence.

## Materials and methods

### Protein production for X-ray crystallography

#### RpfR_Ct_(FI)

*rpfR*_*Ct*_(FI) (amino acids 1–95) was PCR amplified from synthetic full-length *C*. *turicensis* z3032 *rpfR* (codon optimized for *E*. *coli* expression, Genscript) using Phusion High-Fidelity DNA Polymerase (New England Biolabs) and the oligonucleotide pair primer 1 and primer 2. The amplified insert was analyzed using a 0.1% agarose gel, and the band corresponding to the insert was excised and gel purified. Gibson Assembly (New England Biolabs) was used to integrate the purified insert into pTB146 [49] and linearized using the restriction enzymes SapI and XhoI to generate the vector pHis6-SUMO-RpfR_Ct_(FI).

pHis6-SUMO-RpfR_Ct_(FI) was transformed and overexpressed in *E*. *coli strain* BL21(DE3) by growing cells at 37 °C and 200 RPM in LB medium containing 100 μM ampicillin until OD_600_ = 0.9. Cells were induced using 500 μM isopropyl β-D-1-thiogalactopyranoside (IPTG) and grown for a further 20 h at 18 °C and 200 RPM. Cells were then pelleted at 7,000 × g for a period of 15 min.

Pelleted cells were resuspended in buffer A (500 mM NaCl, 50 mM HEPES [pH 7.5], 40 mM imidazole, 10 μg/mL DNAse, and 1 mM phenylmethanesulfonyl fluoride [PMSF]). The cells were lysed by two passages through a cell disruptor, and the cell lysate was subsequently clarified at 35,000 × g for a period of 45 min at 4 °C. Clarified lysate was passed over His60 Superflow Ni Resin (Takara Bio, United States of America) equilibrated in buffer A. The resin was washed with buffer A and was eluted with an increasing step gradient of imidazole in buffer A. Fractional purity was evaluated using SDS-PAGE. The highest purity fractions were pooled and dialyzed overnight against buffer B (100 mM NaCl, 50 mM HEPES [pH 7.5]). His_6_-Ulp1 SUMO protease was added to the pooled fractions prior to dialysis to cleave the His_6_-SUMO affinity tag.

Following cleavage, the dialyzed sample was reapplied to fresh His60 Superflow resin to remove the majority of the His_6_-SUMO tag and His_6_-Ulp1, with the flow-through containing RpfR_Ct_(FI). The flow-through was subsequently diluted using buffer C (50 mM HEPES [pH 7.5]) to a final concentration of 50 mM NaCl and 50 mM HEPES (pH 7.5). The diluted protein was then passed over a MonoQ anion exchange column (GE Healthcare). RpfR_ct_(FI) eluted in the MonoQ column flow-through and was separated from all significant contaminants. Following anion exchange, the pure fractions of RpfR_Ct_(FI) were pooled and concentrated using a 3-kDa molecular weight–cutoff pressure concentrator (Sartorius) and loaded onto a Superdex 200 16/70 column (GE Healthcare) equilibrated with buffer D (100 mM NaCl, 20 mM MOPS [pH 7.0], and 1 mM TCEP). Following size exclusion chromatography, RpfR_ct_(FI) was concentrated to 7.9 mg/mL (A_280_ 0.1% [= 1 g/L] = 1.353, calculated using the ExPASy ProtParam server [50]) using a 3-kDa cutoff Vivaspin concentrator and stored at −80 °C.

Selenomethionyl (SeMet) RpfR_Ct_(FI) was produced by following a modified version of a published protocol for incorporating selenomethionine into proteins [51]. BL21(DE3) cells were transformed with pHis6-SUMO-RpfR_Ct_(FI) and grown in minimal media containing 100 μM ampicillin at 37 °C and 200 RPM until OD_600_ = 0.76. Using sterile technique, the cells were pelleted at 3,000 RPM for 15 min, and the minimal media supernatant was removed. Cells were washed twice with a buffer containing 10 g/L K_2_HPO_4_ and 1 g/L sodium acetate. Cells were then resuspended in fresh minimal media containing 100 μM ampicillin and 50 mg/L selenomethionine in place of methionine. Cells were induced with 500 μM IPTG and grown for an additional 15.5 h at 18 °C and 200 RPM. Cells were pelleted at 7,000 × g for 15 min. The purification of SeMet RpfR_Ct_(FI) was identical to that of native protein.

#### RpfR_Ct_(FI)–RpfF_Bc_

Full-length *rpfF*_*Bc*_ (amino acids 1–287) was amplified using Phusion High-Fidelity DNA Polymerase and WT *B*. *cenocepacia* HI2424 genomic DNA with the oligonucleotide pair primer 3 and primer 4. The gel purified insert was cloned into pBB75 that had been linearized using NdeI and EcoRI. Gibson Assembly was then used to generate the construct, pRpfF_Bc_.

pHis6-SUMO-RpfR_Ct_(FI) and pRpfF_Bc_ were cotransformed into BL21(DE3) cells and grown to OD_600_ = 0.9 at 37 °C and 200 RPM in LB medium containing 100 μM ampicillin and 30 μM kanamycin, after which expression of both proteins was induced by the addition of 500 μM IPTG. Following induction, cells were grown for an additional 11 h at 25 °C and 200 RPM and pelleted by centrifugation at 7,000 × g for 15 min.

Cells were resuspended in buffer E (300 mM NaCl, 50 mM HEPES [pH 8.0], 20 mM imidazole, 5% [vol/vol] glycerol, 10 μg/mL DNAse, and 1 mM PMSF) and lysed by two passages through a French press at approximately 25,000 PSI. Lysate was clarified at 35,000 × g for 45 min at 4 °C. Clarified lysate was applied to His60 Superflow resin equilibrated with buffer E. The resin was then washed with buffer E containing 40 mM imidazole. His_6_-SUMO-RpfR_ct_(FI) coeluted in a 1:1 stoichiometric amount with RpfF_Bc_ following an increasing step gradient of imidazole in buffer E. Fractional purity was evaluated by SDS-PAGE, and the highest purity fractions were combined and dialyzed overnight against buffer F (100 mM NaCl, 50 mM HEPES [pH 8.0], and 5% [vol/vol] glycerol) in the presence of His_6_-Ulp1 SUMO protease. The His_6_-SUMO tag and His_6_-Ulp1 were subsequently removed by passing the dialyzed sample over fresh His60 Superflow resin equilibrated with buffer F. Pure RpfR_Ct_(FI)–RpfF_Bc_ complex was concentrated using a 10-kDa cutoff filter (Sartorius) and loaded onto a Superdex 200 16/70 column equilibrated with buffer G (100 mM NaCl, 20 mM HEPES [pH 8.0], 1% [vol/vol] glycerol, and 1 mM TCEP). Fractions of the complex were subsequently combined and concentrated to 3.35 mg/mL (as evaluated by the Bradford method) using a 10-kDa cutoff Vivaspin concentrator.

#### RpfR_Ct_(PAS)

*rpfR*_*Ct*_(PAS) (amino acids 115–224) was PCR amplified from synthetic full-length *C*. *turicensis* z3032 *rpfR* using Phusion High-Fidelity DNA Polymerase and the oligonucleotide pair primer 5 and primer 6. Gibson Assembly was used to insert the gel purified insert into XhoI/SapI linearized pTB146, generating vector pHis6-SUMO-RpfR_Ct_(PAS). His_6_-SUMO-RpfR_Ct_(PAS) was overexpressed in BL21(DE3) cells transformed with pHis6-SUMO-RpfR_Ct_(PAS) grown to OD_600_ = 0.60 at 37 °C and 220 RPM and then moved to 18° C and 200 RPM. When the OD_600_ reached 0.90, expression was induced with 500 μM IPTG, and cells were grown for an additional 18 h at 18 °C and 200 RPM. Following induction, cells were harvested by pelleting at 7,000 × g for 15 min.

Cell pellets were resuspended in buffer H (1 M NaCl, 50 mM HEPES [pH 7.0], 20 mM imidazole, 10 μg/mL DNAse, and 1 mM PMSF). Cells were lysed following two passages through a cell disruptor, and the lysate was clarified at 35,000 × g for a period of 45 min at 4 °C. The lysate was passed over His60 Superflow Ni resin equilibrated in buffer H and was washed using buffer H. RpfR_Ct_(PAS) was eluted using buffer H containing increasing concentrations of imidazole. Fractional purity was assessed using SDS-PAGE. The highest purity fractions were pooled and combined with His_6_-Ulp1 SUMO protease. The protease reaction was dialyzed overnight against buffer H absent imidazole. The dialyzed fraction was passed over fresh His60 Superflow resin to remove His_6_-Ulp1 and the His_6_-SUMO tag. The pure RpfR_Ct_(PAS) in the flow-through was subsequently concentrated using a 3-kDa Vivaspin cutoff filter and loaded onto a Superdex 200 16/70 column equilibrated with buffer I (100 mM NaCl, 20 mM HEPES [pH 7.0], and 1 mM TCEP). Following size exclusion chromatography, RpfR_ct_(PAS) was concentrated to 4.7 mg/mL (as evaluated by the Bradford method) using a 3-kDa cutoff Vivaspin concentrator and stored at −80 °C.

The RpfR_ct_(PAS)–BDSF complex was obtained by coexpressing His_6_-SUMO-RpfR_Ct_(PAS) and full-length RpfF_Bc_ (amino acids 1–287) using BL21(DE3) cells cotransformed with plasmids pHis6-SUMO-RpfR_Ct_(PAS) and pRpfF_Bc_. Cells were grown at 37 °C and 220 RPM in LB medium containing 100 μM ampicillin and 30 μM kanamycin until OD_600_ = 0.9. The temperature was then decreased to 18 °C and expression was induced with 100 μM IPTG. Cells were grown for an additional 16 h prior to harvesting. All subsequent purification steps of the RpfR_Ct_(PAS)–BDSF complex were identical to the purification of the RpfR_Ct_(PAS)–C12:0 complex, as described above.

### Crystallization and diffraction data collection

#### RpfR_Ct_(FI)

Crystals of RpfR_Ct_(FI) were obtained by the hanging drop vapor diffusion method at 20 °C. A 1:1 mixture of protein solution (7.9 mg/mL RpfR_Ct_[FI], 1 mM BDSF, and 0.62% [vol/vol] DMSO) and mother liquor solution (75 mM NaH_2_PO_4_ and 20% [vol/vol] PEG 3350) was set up above a well containing 700 μL mother liquor solution. Crystals appeared overnight and were cryoprotected by soaking into a solution of 75 mM NaH_2_PO_4_, 20% (vol/vol) PEG 3350, 15% (vol/vol) glycerol, 1 mM BDSF, and 0.62% (vol/vol) DMSO. Crystals grew in the presence or absence of 1 mM BDSF (Sigma-Aldrich) and 0.62% (vol/vol) DMSO. SeMet derived crystals were obtained by mixing SeMet-RpfR_Ct_(FI) (6.7 mg/mL) with mother liquor solution (100 mM NaH_2_PO_4_ and 17.5% [vol/vol] PEG 3350) in a 1:1 ratio over a well containing 700 μL mother liquor solution. Crystals were cryoprotected using 100 mM NaH_2_PO_4_, 17.5% (vol/vol) PEG 3350, and 15% (vol/vol) glycerol.

Data were collected at the Stanford Synchrotron Radiation Lightsource (SSRL) beamline 14–1 using a MARmosaic 325 CCD detector and processed using the HKL software package [52]. Initial phases were calculated with the SAD method using PHENIX (AutoSol) [53]. AutoSol located the position of the individual Se atoms and built an initial mode to 1.1 Å resolution with a 0.554 figure of merit. PHENIX(AutoBuild) [54] was used to further build and refine the model, followed by subsequent manual model building in COOT [55] and refinement using PHENIX [56]. The completed SeMet model was refined into data collected from an isomorphic native crystal of RpfR_Ct_(FI) to a resolution of 1.2 Å. Early rounds of refinement included simulated annealing, individual B-factors, real space, rigid body, and individual atomic coordinate refinement. In later rounds, waters and hydrogens were included, and ADP weighting was optimized.

#### RpfR_Ct_(PAS)–C12:0

Crystals of RpfR_Ct_(PAS) bound to lauric acid were grown using the sitting drop vapor diffusion method at 20 °C. An RpfR_Ct_(PAS) protein solution (4.7 mg/mL RpfR_Ct_[PAS],1 mM BDSF, and 0.62% [vol/vol] DMSO) was combined 1:1 with the mother liquor solution (200 mM sodium formate, 18% [vol/vol] PEG 3350, and 100 mM HEPES [pH 7.75]) and allowed to equilibrate above a well containing 700 μL mother liquor solution. Crystals were cryoprotected in a solution containing 200 mM sodium formate, 20% (vol/vol) PEG 3350, 100 mM HEPES (pH 7.75), 1 mM BDSF, 0.62% (vol/vol) DMSO, and 15% (vol/vol) glycerol. Data were collected at SSRL beamline 9–2 using a Dectris Pilatus 6M detector and processed using the HKL software package [52].

Initial phases for RpfR_Ct_(PAS)-lauric acid were determined in Phaser [57] using the molecular replacement method and the N-terminal PAS domain of PpANR MAP3K from *Physcomitrella patens* (PDB:5IU1) [58] as a search model. The initial model building and refinement were performed using PHENIX (AutoBuild) [54] with three rounds of simulated annealing to remove phase bias from the starting model. Further manual building was performed using COOT [55] and refined in PHENIX [56]. Individual atomic coordinates, real-space, and individual B-factors were refined during the early stages of model building. As the model improved, excess difference density that did not resemble water or sidechain density became prominent. C12:0 was successfully built into the excess difference density. During the final stages of refinement, TLS parameters (generated in PHENIX [59]) and ADP-optimized weights were included in the refinement, and hydrogens were added to the model. While some electron density corresponding to residues 115 and 207–212 was evident, attempts to build and refine residues into this disordered region of the map were unsuccessful.

#### RpfR_Ct_(PAS)–BDSF

Crystals of RpfR_Ct_(PAS)–BDSF were produced by the hanging drop vapor diffusion method at 20 °C by mixing RpfR_Ct_(PAS)–BDSF (4.7 mg/mL) with mother liquor solution (150 mM lithium citrate and 16% [vol/vol] PEG 3350) in a 1:1 ratio above a well containing 700 μL mother liquor solution. Crystals were cryoprotected by soaking in 150 mM lithium citrate, 16% (vol/vol) PEG 3350, and 15% (vol/vol) glycerol.

Data were collected on a home source Rigaku Micro/Max-007HF Rotating Copper Anode X-ray Generator using a Rigagku RAXIS-IV++ detector and processed with the HKL software package [52]. Phaser [57] was used to obtain initial phases of RpfR_Ct_(PAS)–BDSF by using the previously determined structure of RpfR_Ct_(PAS)–C12:0 (PDB:6DGG) as a search model. In order to reduce the effect of phase bias on ligand density, C12:0 and all water molecules were removed from the search model, and three rounds of simulated annealing were performed during the initial stages of refinement to remove any phase bias toward ligand identity. Individual atomic coordinates, real-space, rigid body, and individual B-factors were initially refined, with TLS parameters (generated in PHENIX [59]) and individual ADP weights added during later rounds of refinement. Midway through refinement, both C12:0 and BDSF were built into the model and refined separately. BDSF and its accompanying restraints were generated using PHENIX (eLBOW) [60] and assigned the three letter code GEY. While both ligands fit the difference density in the RpfR_Ct_(PAS) ligand-binding pocket, C12:0 adopted the synperiplanar configuration of BDSF following refinement. BDSF was included in the model for the duration of the refinement. Near the end of the refinement, hydrogens were included in the model. Insufficient electron density was available to build the following residues: 115 and 206–212. As with the C12:0 complexed structure, while electron density was present for residues 206–212, all attempts to build productively into the density were unsuccessful.

#### RpfF_Bc_–RpfR_Ct_(FI)

Crystals of the RpfF_Bc_–RpfR_Ct_(FI) complex were grown using the hanging drop vapor diffusion method at 20 °C by mixing RpfF_Bc_–RpfR_Ct_(FI) (3.35mg/mL) with mother liquor solution (100 mM sodium acetate [pH 5.6] and 250 mM ammonium phosphate dibasic) in a 1:2 ratio above a well containing 700 μL mother liquor solution. Crystals were cryoprotected by soaking in a solution of 100 mM sodium acetate (pH 5.6), 250 mM ammonium phosphate dibasic, and 30% (vol/vol) glycerol. Data were collected at SSRL beamline 9–2 using a Dectris Pilatus 6M detector and processed using the HKL software package [52].

Initial phases were obtained by molecular replacement using Phaser [57], with the structures of RpfR_Ct_(FI) (PDB:6DGA) and *B*. *cenocepacia* RpfF (PDB:5FUS) as search models [17]. In the early stages of model building, simulated annealing, rigid-body, individual atomic coordinates, individual B-factors, and real-space refinement were performed in PHENIX [56]. As refinement proceeded, rigid-body refinement and simulated annealing were discontinued, while TLS parameters (generated in PHENIX [59]), ADP-optimized weights, and waters were included. There was insufficient electron density to build the following residues: RpfF_Bc_ 1, 278–287, and RpfR_Ct_(FI) 1–4. During the later stages of refinement, hydrogens were added to the model. Additionally, four molecules of glycerol and one molecule of phosphate were built into their corresponding electron density.

### Protein production for RpfR(FI)–RpfF enzymatic assay

#### AasS

*V*. *harveyi aasS*, codon optimized for expression in *E*. *coli*, was synthesized and inserted into pET15-b expression vector containing an amino-terminal His_6_-tag to create pAasS (Genscript). Following methods previously described [61,62], pAasS was transformed into chemically competent BL21(DE3) cells and grown to OD_600_ = 0.6 at 37 °C and 200 RPM in LB medium containing 100 μM ampicillin, at which point the cells were moved to 25 °C and 200 RPMS and induced using 1 mM IPTG when they reached OD_600_ = 0.9. Cells were further grown for an additional 20 h and harvested following centrifugation at 7,000 × g for 15 min.

The cell pellet was resuspended in Buffer J (300 mM NaCl, 20 mM Tris-HCl [pH 7.5], 10% [vol/vol] glycerol, and 10 mM imidazole) and lysed by two passes through a French press at approximately 25,000 PSI. Cell lysate was clarified at 35,000 × g at 4 °C for 40 min. Clarified lysate was applied to His60 Superflow resin equilibrated with Buffer J. The resin was washed with Buffer J containing 40 mM imidazole. His_6_-AasS was eluted using an increasing gradient of imidazole in Buffer J and analyzed using SDS-PAGE. The purest fractions were combined and dialyzed into the final AasS storage buffer, Buffer K (20 mM Tris [pH 7.5], 10% [vol/vol] glycerol, 1 mM EDTA, 0.1 mM DTT, and 0.002% [vol/vol] Triton X-100). Dialyzed AasS was concentrated to 3.07 mg/mL (A_280_ 0.1% [= 1 g/L] = 1.05 calculated with the ExPASy ProtParam server [50]), with a 30-kDa cutoff Vivaspin concentrator and stored at −80 °C.

#### RpfR_Bc_(FI)

*B*. *cenocepacia rpfr*_*Bc*_*(FI)* (amino acids 1–94) was amplified using Phusion High-Fidelity DNA Polymerase from WT *B*. *cenocepacia* HI2424 genomic DNA and the oligonucleotide pair primer 7 and primer 8. The PCR amplified insert was gel purified and cloned into a SapI/XhoI linearized pTB146 vector using the Gibson Assembly to generate pHis6-SUMO-RpfR_Bc_(FI).

pHis6-SUMO-RpfR_Bc_(FI) was transformed into BL21(DE3) cells and grown to OD_600_ = 0.70 in LB containing 100 μM ampicillin at 37 °C and 200 RPM then moved to 18 °C and 200 RPM. Upon reaching OD_600_ = 0.9, expression was induced with 500 μM IPTG. Cells were grown for an additional 17 h at 18 °C and 200 RPM and harvested by centrifugation at 7,000 × g for 15 min. RpfR_Bc_(FI) was purified identically to RpfR_Ct_(FI) (see above) and concentrated to a 549 μM (extinction coefficient = 11,000 M^−1^ cm^−1^ calculated with the ExPASy ProtParam server [50]).

#### RpfF_Bc_

Full-length *rpfF*_*Bc*_ (amino acids 1–287) was amplified with Phusion High-Fidelity DNA Polymerase from WT *B*. *cenoecacia* HI2424 genomic DNA and the oligonucleotide pair primer 9 and primer 10. The PCR insert was gel purified and cloned into a SapI/XhoI linearized pTB146 vector using the Gibson Assembly to generate pHis6-SUMO-RpfF_Bc_. pHis6-SUMO-RpfF_Bc_ was transformed into BL21(DE3) cells and grown in LB medium containing 100 μM ampicillin and supplemented with 0.2% (vol/vol) glucose. Cells were grown to an OD_600_ = 0.78 at 37 °C and 220 RPM. Cells were then moved to 25 °C and 200 RPM and induced with 500 μM IPTG upon reaching OD_600_ = 0.93. Following induction, cells were grown for an additional 20 h and harvested by pelleting at 7,000 × g for 10 min.

His_6_-SUMO-RpfF_Bc_ was purified using a protocol similar to that previously reported [17], with the following modifications. Cells were resuspended in buffer L (300 mM NaCl, 50 mM Na phosphate [pH 8.0], 5% [vol/vol] glycerol, 10 mM imidazole, and 10 μg/mL DNAse) and passed two times through a cell disruptor. Lysate was clarified at 35,000 × g for 40 min at 4 °C. Clarified cell lysate was incubated overnight with gentle rocking in the presence of His60 Superflow resin equilibrated in buffer L at 4 °C. The resin was packed into a column and washed using 40 mM imidazole in buffer L. His_6_-SUMO-RpfF_Bc_ was eluted from the resin using a step gradient of 100, 250, and 500 mM imidazole in buffer L. The purest fractions were combined and dialyzed overnight in the presence of His_6_-Ulp1 SUMO protease into buffer M (100 mM NaCl, 50 mM Na phosphate [pH 8.0], and 5% [vol/vol] glycerol) to remove imidazole. The dialyzed protein was incubated with fresh His60 Superflow resin for 1 h at 4 °C with gentle rocking to remove the His_6_-SUMO tag and His_6_-Ulp1. The pure RpfF_Bc_ that flowed through the column was concentrated using a 10-kDa cutoff pressure concentrator to 1.71 mg/mL (evaluated using the Bradford method) and loaded onto a Superdex 200 16/70 column equilibrated with buffer N (100 mM NaCl, 50 mM Na phosphate [pH 8.0], 5% [vol/vol] glycerol, and 1 mM TCEP). Peak fractions were analyzed with SDS-PAGE and combined to a final concentration of 6.4 μM (extinction coefficient = 36,900 M^−1^ cm^−1^ calculated with the ExPASy ProtParam server [50]). Aliquots of RpfF_Bc_ were stored at −80 °C.

### *acpP* and *acpS* cloning

Obtaining holo-ACP_Ec_ (ACP_Ec_ with the 4′-phosphopantetheinyl prosthetic group on serine 36) requires ACP to be coexpressed alongside the *E*. *coli* Acyl Carrier Protein Synthase (AcpS_Ec_). To coexpress ACP_Ec_ and AcpS_Ec,_ we engineered a construct similar to the one previously described in [63], by cloning *acpP*_*Ec*_ and *acpS*_*Ec*_ into pQlink-H and pQlink-N, respectively [64]. pQlink-H was chosen in order to facilitate ACP_Ec_ purification using a TEV protease cleavable His_7_-tag. Both inserts were amplified from *E*. *coli MG1655* genomic DNA using Phusion High-Fidelity DNA Polymerase. The *acpP*_*Ec*_ insert was generated using the oligonucleotide pair primer 11 and primer 12. The *acpS*_*Ec*_ insert was amplified using the oligonucleotide pair primer 13 and primer 14. Amplified and gel purified *acpP*_*Ec*_ was inserted into BamHI and NotI linearized pQlink-H using the Gibson Assembly to generate plasmid pAcpP_Ec_-His7. Likewise, amplified and gel purified *acpS*_*Ec*_ was inserted into BamHI and NotI linearized pQlink-N, generating pAcpS_Ec_.

A single-expression construct for both proteins was generated by digesting pAcpS_Ec_ with PacI and pAcpP_Ec_-His7 with SwaI. Following heat inactivation of the restriction digests at 65 °C for 20 min, both reactions were incubated with LIC-qualified T4 DNA polymerase (Novagen) in the presence of either dCTP (pAcpS_Ec_ digest) or dGTP (pAcpP_Ec_-His7 digest) at 25 °C for 30 min. The T4-treated DNA fragments were then combined in a 1:1 ratio and annealed at 65 °C for five min, followed by a 2 min incubation on ice and a 15 min incubation at room temperature. Following the addition of EDTA to a final concentration of 1.25 mM, the annealing reactions were transformed into Lucigen SOLO hypercompetent cells (Lucigen), generating pHis7-AcpP_Ec_/N-AcpS_Ec_.

### Holo-ACP purification

Purification of holo-ACP_Ec_ was accomplished using several modified protocols [62,63,65]. pHis7-AcpP_Ec_/N-AcpS_Ec_ was transformed into C41(DE3) chemically competent *E*. *coli* cells and grown up in LB medium containing 100 μM ampicillin. Cells were grown to OD_600_ = 0.6 at 37 °C and 220 RPM and induced with 1 mM IPTG. Following induction, cells were grown for an additional 22 h at 18 °C and 200 RPM and then pelleted at 7,000 × g for 15 min. The pellet was resuspended in lysis buffer O (500 mM NaCl, 50 mM Tris [pH 8.8], 10 mM MgCl_2_, 5 mM *β*-mercaptoethanol, 20 mM imidazole, and 10 μg/mL DNAse). Cells were lysed by two passages through a cell disruptor and clarified at 35,000 × g for 40 min at 4 °C. Clarified lysate was incubated with His60 Superflow resin equilibrated in buffer O for 1.5 h with gentle rocking at 4 °C. The resin was washed once with buffer O and a second time with buffer O containing 40 mM imidazole. His_7_-ACP_Ec_ copurified with AcpS_Ec_ following elution with increasing concentrations of imidazole in buffer O. Fractional purity was analyzed with SDS-PAGE. The purest fractions were combined with His_6_-TEV protease. EDTA was added to a final concentration of 50 mM, and the reaction was incubated at 4 °C overnight. The overnight reaction was then dialyzed against buffer P (50 mM NaCl, 25 mM MOPS [pH 7.1], and 1mM *β*-mercaptoethanol) to remove imidazole. Dialyzed fractions were incubated with fresh His60 Superflow resin equilibrated in buffer P and allowed to rock at 4 °C for 2 h to remove the His_7_-tag and His_6_-TEV protease. The flow-through from the resin was loaded onto a Q Sepherase Fast Flow Column (Pharmacia) equilibrated in buffer P. Holo-ACP_Ec_ was successfully separate from AcpS_Ec_ using an increasing gradient of KCl in buffer P. The purest holo-ACP_Ec_ fractions were combined and concentrated using a 3-kDa cutoff filter and loaded onto a Superdex 200 16/70 column equilibrated in buffer Q (100 mM NaCl, 20 mM Tris [pH 7.5], and 0.87 mM TCEP). Peak fractions were analyzed using SDS-PAGE and concentrated to 444.4 μM (extinction coefficient = 1,800 M^−1^ cm^−1^ [Sigma-Aldrich]) using a 3-kDa Vivaspin concentrator.

### ACP-charging reaction

Optimal charging reaction conditions were based on those previously reported [15,16,63]. C12:0–ACP_Ec_ was charged with substrate by incubating 142 μM holo-ACP_Ec_ with 2.5 mM C12:0, 10 mM ATP, 2 μM AasS, in ACP-charging buffer R (100 mM Tris [pH 7.8], 10 mM MgCl_2_, and 1 mM TCEP) for 4 h at 37 °C. Following charging, the reaction was precipitated by the addition of two equivalent reaction volumes of acetone and incubated at −20 °C overnight. The precipitated protein was pelleted at 20,000 × g for 30 min. The supernatant was removed, and the pellet was washed two times with two additional reaction volumes of acetone. The pellet was then air dried and dissolved in 20 mM Tris (pH 7.5) to a final concentration of 111 μM charged-ACP_Ec_.

### RpfF thioesterase assay

The thioesterase reaction conditions were chosen based on previously published methodologies [15,16]. RpfF thioesterase activity was measured using a 10-μL reaction consisting of 78 μM C12:0–ACP_Ec_ substrate, 0.64 μM RpfF_Bc_ (or control buffer N), and either 6.4 μM RpfR_Bc_(FI), 1.28 μM RpfR_Bc_(FI), or control buffer D in the thioesterase assay buffer (100 mM Tris [pH 7.5]). This reaction was incubated at 37 °C for 30 min and then heat inactivated at 95 °C for 2 min. Reactions were analyzed with a conformation-sensitive nondenaturing gel containing 20% polyacrylamide, 375 mM Tris (pH 8.8), and 2.5 M urea. Gels were stained with Coomassie Brilliant Blue, and band intensities were measured using a LI-COR Odyssey CLx Imager System (LI-COR Biosciences) and quantified using Image Studio version 3.1 (LI-COR Biosciences).

#### Production of RpfR_Bc_(FI-PAS)-RpfF_Bc_

*B*. *cenocepacia rpfr*_*Bc*_(FI-PAS) (amino acids 1–223) was amplified with Phusion High-Fidelity DNA Polymerase from WT *B*. *cenoecacia* HI2424 genomic DNA and the oligonucleotide pair primer 7 and primer 15. The PCR-amplified insert was gel purified and cloned into a SapI/XhoI linearized pTB146 vector using the In-Fusion method (Clonetech) to generate pHis6-SUMO-RpfR_Bc_(FI-PAS).

pHis6-SUMO-RpfR_Bc_(FI-PAS) and pRpfF_Bc_ were cotransformed into BL21(DE3) cells and grown at 37 °C and 200 RPM to OD_600_ = 0.7 in LB medium containing 100 μM ampicillin and 30 μM kanamycin. At this point, the growth temperature was decreased to 25 °C. Upon reaching OD_600_ = 0.9, expression of both proteins was induced by the addition of 500 μM IPTG. Following induction, cells were grown for an additional 17 h at 25 °C and 200 RPM and pelleted by centrifugation at 7,000 × g for 15 min.

Cells were resuspended in buffer S (300 mM NaCl, 50 mM HEPES [pH 8.0], 20 mM imidazole, 10% [vol/vol] glycerol, 10 μg/mL DNAse, and 1 mM PMSF) and lysed by two passages through a French press at approximately 25,000 PSI. Lysate was clarified at 35,000 × g for 45 min at 4 °C. Clarified lysate was applied to His60 Superflow resin equilibrated with buffer S. The resin was then washed with buffer S containing 40 mM imidazole. His_6_-SUMO-RpfR_Bc_(FI-PAS) coeluted in a 1:1 stoichiometric amount with RpfF_Bc_ following an increasing step gradient of imidazole in buffer S.

### Genetic engineering of *B*. *cenocepacia*

Isogenic mutations were created using methods described by Fazli and colleagues [66]. For single-gene deletions, approximately 1,000 bp upstream and downstream of the target gene were amplified using high-fidelity PCR and joined using single overlap extension PCR, using the following conditions: 98 °C for 2 min; 3 cycles of 98 °C for 15 s, 64 °C for 30 s, 72 °C for 1 min; 72 °C for 1 min. After the addition of standard attB1 and attB2 primers, a second round of PCR was performed, using the following conditions: 98 °C for 2 min; 27 cycles of 98 °C for 15 s, 64 °C for 30 s, 72 °C for 2 min; 72 °C for 7 min. This approximately 2,000 bp fragment was then gel purified and inserted into a pDONPREX18Tp-SceI-PheS plasmid containing a trimethoprim (Tp) resistance cassette using Gateway cloning. For single-nucleotide mutations, the target gene was cloned into the same plasmid and site-directed mutagenesis was used to create the intended point mutation. The resulting gene-replacement vectors were electroporated into competent DH5α *E*. *coli* and introduced by conjugation into *B*. *cenocepacia* via triparental mating. Matings were performed by combining 200 μL of overnight cultures of the DH5α strain containing the gene replacement vector, 200 μL of S.17 *E*. *coli* containing the conjugation helper vector pEVS104, and 50 μL of the recipient *B*. *cenocepacia* strain into a cell pellet, resuspending the pellet in 30 μL of 10 mM MgSO_4_ and spotting onto tryptic soy agar (TSA) plates. The cell mixture was scraped from the plate after 24 h at 37 °C using an inoculating loop, resuspended in 1 mL of PBS, and plated at different dilutions onto Vogel–Bonner minimal medium (VBMM) agar containing 100 μg/mL Tp. Four Tp-resistant colonies were selected and grown first in tryptic soy broth, followed by VBMM broth containing 0.1% chlorophenylalanine before being plated onto four TSA plates. 100 colonies from each plate were picked using pipette tips and patched onto both TSA and TSA-Tp100. Four candidates for each mutation were selected by sensitivity to Tp and sequenced using whole genome sequencing according to Baym and colleagues [67] on an Illumina NextSeq 500 to a minimum average of 30x coverage. The correctly made mutants were confirmed to be otherwise isogenic using the variant calling program Breseq v. 0.31 [68].

### Quantification of fitness in the biofilm life cycle

Freezer stocks were revived overnight and then grown for 24 h in M9 minimal medium + 3% galactose (GMM) at 37 °C. Equal volumes of each competitor were added to 3% GMM containing three 7 mm polystyrene beads, and a planktonic sample of that mixture was serially diluted and plated on half-strength T-soy X-gal plates to enumerate starting CFU/mL. Samples taken from a bead at subsequent 12-h time intervals over 48 total h were serially diluted and plated in half-strength T-soy X-gal plates to enumerate CFU/bead. Fitness was calculated as the difference in Malthusian parameters between the mutant and wild type ancestor in units of time^−1^, as follows: ln (*Nm*1/*Nm*0) − ln (*N*_WT_1/*N*_WT_0), in which *N* is cell number and *m* is mutant and WT is wild type at time 0 or 1 (e.g., *Nm*1 is the number of mutant cells at time 1) [69].

### Quantification of BDSF in *B*. *cenocepacia* supernatants

Four independent cultures of each strain (WT, Δ*rpfR*, Δ*rpfF*, and Δ*FI*) were inoculated into 2 mL Luria broth (LB) in test tubes from frozen stocks and grown at 35 °C, 200 RPM overnight (approximately 14 h). The next day, cultures were diluted 1:500 into 10 mL fresh LB in 50 mL flasks in quadruplicate and grown at 35 °C, 200 RPM for 12 h. After growth, 1 mL of culture was removed from each flask and placed into 2 mL tubes and 20 μL of culture was used to measure cell density by dilution plating. The cultures were centrifuged at max speed (15,000 × g) at room temperature for 1 min. Supernatants were removed and placed into new 2-mL tubes, and the solutions were acidified using 12 M HCl until the pH was less than 4.0. Three hundred μL of ethyl acetate was added to each sample, and the tubes were vortexed for 5 min. The samples were then centrifuged for 10 min at 8,000 × g at room temperature to separate organic and aqueous phases. The top (organic) phase was removed and placed into new 1.5-mL tubes. The resulting organic solution was evaporated using a heated vacuum centrifuge, and the dried pellet was resuspended in 100 μL 1:1 methanol/water, placed in mass spectrometry vials, and analyzed by liquid chromatography-mass spectrometry on a Xevo TQ-D Triple Quadruple mass spectrometer (Waters) coupled with an UPLC system (Acquity, model BSM).

Liquid chromatography separation was carried out on an Acquity UPLC BEH reverse-phase column (1.7 μm, 2.1 mm × 150 mm, Waters). Solvent A was 10 mM ammonium formate in water. Solvent B was 100% methanol. 10 μL of sample was autoinjected into the column and subjected to solvent A and B gradients as follows: t = 0 minutes, 10% solvent B; t = 4 minutes, 98% solvent B; t = 7.01 minutes, 10% solvent B at a flow rate of .200 mL/min for a total of 10 min per sample. Under these conditions, BDSF had a retention time of 5.6 min. BDSF was detected in selected reaction monitoring (SRM) in negative ionization mode following the *m/z* 198 → 197 at 30 eV. The signal for BDSF in biological samples was defined as the observed peak area on the chromatography trace determined by the MassLynx software (Waters). Chemically synthesized BDSF (Adipogen, San Diego, CA) was used to determine retention time and optimize fragmentation patterns.

### Quantification of c-di-GMP in *B*. *cenocepacia* biofilm cells

Freezer stocks were revived overnight in T-soy broth and then grown for 24 h in M9 minimal medium + 3% galactose (GMM) at 37 °C. 1.25 mL of each strain was added to 125 mL 3% GMM in a flask containing 100 7-mm polystyrene beads and incubated at 100 rpm at 37 °C for 12 h. While harvesting, flasks were incubated on ice for 10 min. For the biofilm phase, the planktonic culture was discarded, and the beads were washed with 60 mL of cold PBS. These were then divided into four 50 mL centrifuge tubes containing 20 mL of cold PBS each. Each tube was vortexed for 30 s to remove the attached cells, and the PBS from all four sets was combined. The samples were then serially diluted and plated in half-strength T-soy X-gal plates to enumerate CFU/flask and then centrifuged at max speed for 15 min at 25 °C. Pellets were then resuspended in 500 μL of ice-cold extraction buffer (methanol:acetonitrile:dH_2_O 40:40:20 + 0.1 N formic acid). The suspensions were transferred to 1.5-mL microfuge tubes and incubated at −20 °C for 1 h, followed by 95 °C for 10 min. The tubes were then centrifuged to pellet the cell debris. 400 μL of the liquid phase was transferred to another microfuge tube and 16 μL of neutralization buffer (15% ammonium bicarbonate) was added. The tubes were stored at −80 °C. Quantification of c-di-GMP using mass spectroscopy was then carried out, as previously described [70].

### Quantification of c-di-GMP in *B*. *cenocepacia* planktonic cells

Freezer stocks were revived overnight in T-soy broth and then grown for 24 h in M9 minimal medium + 3% galactose (GMM) at 37 °C. 1.25 mL of each strain was added to 125 mL 3% GMM and incubated at 100 rpm at 37 °C for 12 h. While harvesting, flasks were incubated on ice for 10 min. The samples were then serially diluted and plated in half-strength T-soy plates to enumerate CFU/flask and then centrifuged at max speed for 15 min at 4 °C. Pellets were then resuspended in 500 μL of ice-cold extraction buffer (methanol:acetonitrile:dH_2_O 40:40:20 + 0.1 N formic acid). The suspensions were transferred to 1.5-mL microfuge tubes and incubated at −20 °C for 1 h, followed by 95 °C for 10 min. The tubes were then centrifuged to pellet the cell debris. 400 μL of the liquid phase was transferred to another microfuge tube and 16 μL of neutralization buffer (15% ammonium bicarbonate) was added. The tubes were stored at −80 °C. Quantification of c-di-GMP using mass spectroscopy was then carried out, as previously described [70].

### Lipid analysis

Unknown lipids were extracted from RpfR_Ct_(PAS) for analysis, as described previously [17,71]. A 2:1 mixture of chloroform and methanol was added to purified RpfR_Ct_(PAS) in buffer I in a 4:1 ratio. The solution was then mixed vigorously until RpfR_Ct_(PAS) had precipitated. The different phases were allowed to separate, and the chloroform layer was removed for analysis.

Lipid released from purified RpfR_Ct_(PAS) was determined by the analysis of the purified protein extract on an Agilent 6120 LC-MS system equipped with an EMD Millipore Chromolith SpeedROD analytical RP-HPLC column (50 x 4.6 mm). A 0.5 mL solution of RpfR_Ct_(PAS) in buffer I was extracted using a 2-mL mixture of chloroform and methanol (2:1). The bottom organic layer was separated and concentrated using N_2_ flow. The remaining residue was taken in 100 μL of acetonitrile/water (1:1), and the solution was centrifuged to remove any precipitate. 50 μL of the supernatant (or 25 μL of the standards in acetonitrile/water [4:1]) were analyzed by LC-MS using a gradient of 10%–100% acetonitrile in water containing 0.1% formic acid in 10 min at a flowrate of 1 mL/min. The identification of the lipid was determined by the presence of the negative ionization [M-H]^−^ corresponding to either BDSF^−^ (*m/z* calculated for C_12_H_21_O_2_^−^ = 197.2) or C12:0^-^ (*m/z* calculated for C_12_H_23_O_2_^−^ = 199.3) anions as observed in the extracted ion chromatogram of the lipids *m/z*.

Lipid released from purified RpfR_Ct_(PAS) that was coexpressed with RpfF_Bc_, was identified by following the same extraction protocol described above. For LC-MS analysis, a gradient of 40%–100% acetonitrile in water containing 0.1% formic acid in 10 min at a flowrate of 1 mL/min was used to improve the separation between BDSF and C12:0 signals.

### BDSF production assay

Starter cultures of individual strains to be tested were incubated overnight at 37 °C and 200 RPM in 10 g LB (IBI Scientific) per 1 L diH_2_O. In order to remove residual BDSF, starter cultures were pelleted at 15,000 RPM at 25 °C for 10 min. Supernatants were removed from pelleted cells, which were twice resuspended in fresh LB. 25 mL of sterile LB was inoculated with 50 μL of starter culture and grown for 33 h at 37 °C and 200 RPM. Aliquots of the culture were removed at the specified times. To obtain cell-free supernatants, culture samples were centrifuged for 5 min at 15,000 RPM at 25 °C. Supernatants were separated from the pelleted samples and centrifuged for an additional 5 min at 15,000 RPM at 25 °C and stored at −20 °C. Aliquots were diluted and grown on LB agar plates overnight at 37 °C in order to determine the number of colony forming units (CFU) at the specified time.

BDSF production was measured with the *B*. *cenocepacia* H111 pAN-L15 reporter strain using a modified protocol similar to those reported in [20,41]. The reporter strain was grown for 18 h in LB supplemented with 100 μg/mL kanamycin and 80 μg/mL chloramphenicol at 37 °C and 200 RPM to an OD_600_ = 1.6–2.5. The reporter strain was pelleted at 3,000 × g for 30 min at 25 °C and resuspended twice in fresh LB supplemented with 100 μg/mL kanamycin and 80 μg/mL chloramphenicol. 100 μL of reporter strain was added to individual wells of a 96-well flat clear-bottom black polystyrene TC-treated microplate (Costar). Cell-free supernatants from individual donor strain samples were diluted 1:10 (v/v) in fresh LB supplemented with 100 μg/mL kanamycin and 80 μg/mL chloramphenicol. 100 μL of the diluted donor strain cell-free supernatant was added to a well containing 100 μL of the reporter strain and incubated at 30 °C for 22 h. Following incubation, 50 μL decyl aldehyde (Sigma-Aldrich) was added to the lid of the microplate. Samples were shaken for 1 min at 200 RPM prior to measuring cell density (OD_570_), and light counts per second (CPS) for each sample were measured using a Perkin Elmer Envision 2014 plate reader equipped for advanced luminometry to determine relative light units (RLU) for each sample. RLU is defined as CPS/OD_570_. Relative Bioluminescence is defined as RLU/CFU.

### Production of full-length wild-type RpfR_Bc_, RpfR_Bc_–S168A, RpfR_Bc_–N171A, RpfR_Bc_–R186A, and RpfR_Bc_–N201A

Full-length *rpfR*_*Bc*_ (corresponding to amino acids 1–667) was amplified using Phusion High-Fidelity DNA Polymerase from WT *B*. *cenocepacia* HI2424 genomic DNA and the oligonucleotide pair primer 7 and primer 16. The PCR amplified insert was gel purified and cloned into a SapI/XhoI linearized pTB146 vector using In-Fusion (Takara Bio, USA) to generate pHis6-SUMO-RpfR_Bc_(full). Individual BDSF-binding site mutations (S168A, N171A, R186A, and N201A) were introduced into pHis6-SUMO-RpfR_Bc_(full) using the QuikChange II Site Directed Mutagenesis Kit (Agilent Technologies) and the mutagenic primer pairs S168AF, S168AR, N171AF, N171AR, R186AF, R186AR, N201AF, and N201AR (S2 Table).

The full-length wild-type RpfR_Bc_, RpfR_Bc_–S168A, RpfR_Bc_–N171A, RpfR_Bc_–R186A, and RpfR_Bc_–N201A expression vectors were transformed into *E*. *coli strain* BL21(DE3) cells and grown to OD_600_ = 0.60 in LB containing 100 μM ampicillin at 37 °C and 220 RPM, then moved to 25 °C and 200 RPM and induced with 500 μM IPTG. Cells were grown for an additional 16 h at 25 °C and 200 RPM and harvested by centrifugation at 3,000 × g for 30 min at 4 °C. Full-length wild-type RpfR_Bc_, RpfR_Bc_–S168A, RpfR_Bc_–N171A, RpfR_Bc_–R186A, and RpfR_Bc_–N201A were purified in an identical manner. The pelleted cells were resuspended in buffer T (100 mM NaCl, 50 mM MOPS [pH 7.0], 10 μg/mL DNAse, and 1 mM PMSF) and lysed by two passages through a French press at approximately 25,000 PSI. The lysate was clarified at 35,000 × g for 60 min at 4 °C. 100 μL His_6_-Ulp1 was added to 500 μL clarified lysate and incubated at 4 °C for 1 h. Following the incubation, the reaction was centrifuged at 15,000 RPM for 5 min at 25 °C, and the pre- and post-Ulp1 digested samples were evaluated by SDS-PAGE.

## Supporting information

S1 Fig Identification of the lipid molecule released from purified RpfR_Ct_(PAS) as C12:0. The LC-MS ionization traces of (A) total ions and (B) the extracted ion chromatogram of C12:0 in the RpfR_Ct_(PAS) domain protein extract. (C) The mass spectrum corresponding to the C12:0 ion peak showing the negative ionization of the lipid (red arrow). The numerical values underlying panels A and B can be found in S1 Data. C12:0, dodecanoic acid; LC-MS, liquid chromatography–mass spectrometry; PAS, Per-Arnt-Sim; RpfR, regulation of pathogenicity factor R.
(TIF)

S2 Fig Amino acid sequence conservation among RpfR homologues. (A) An amino acid alignment and consensus sequence for conserved residues of the PAS domain from RpfR homologues was generated using CLC Sequence Viewer Version 8 (CLC bio, Aarhus, Denmark). Residues interacting with the carboxylic acid group of either C12:0 or BDSF are highlighted blue. The highly conserved Asn202 that interacts with C3 of BDSF is highlighted in yellow. The residues comprising the hydrophobic acyl–binding pocket are surrounded by unfilled black boxes. The secondary structure representation of RpfR(PAS) was determined using the PyMOL algorithm and is shown below the consensus sequence. (Purple cylinders are α-helices and pink arrows are β-strands. Disordered residues are depicted as a dashed black line.) [72] (B) An amino acid alignment and consensus sequence for conserved residues of the FI domain from RpfR homologues was generated using CLC Sequence Viewer Version 8 (CLC bio, Aarhus, Denmark). Nonconserved residues are marked with asterisks. Residues interacting with RpfF are surrounded by black boxes, with residues forming hydrogen bonds or salt bridges highlighted in blue. Interacting residues were determined by analyzing the RpfR_Ct_(FI)–RpfF_Bc_ structure using the PISA and PDBsum servers [73,74]. The secondary structure representation of RpfR(FI) was determined using the PyMOL algorithm and is shown below the consensus sequence. (Purple cylinders are α-helices and pink arrows are β-strands.) [72] Asn, asparagine; BDSF, *Burkholderia* DSF; C3, carbon 3; C12:0, dodecanoic acid; FI, RpfF interaction; PAS, Per-Arnt-Sim; PDB, Protein Data Bank; PISA, Proteins, interfaces, structures, and assemblies; RpfF, regulation of pathogenicity factor F; RpfR, regulation of pathogenicity factor R.
(TIF)

S3 Fig BDSF is released from purified RpfR_Ct_(PAS) coexpressed with RpfF_Bc_. (A) Ligand released from RpfR_Ct_(PAS) coexpressed with RpfF_Bc_ was analyzed by LC-MS, and the extracted ion chromatograms are shown for the m/z corresponding to BDSF (black curve) and C12:0 (yellow curve). For comparison, a mixture containing both BDSF (200 μM) and C12:0 (200 μM) standards was analyzed by LC-MS, and the extracted ion chromatograms are shown for the m/z corresponding to BDSF (orange curve) and C12:0 (blue curve). (B) The mass spectrum corresponding to the BDSF ion peak showing the negative ionization of the lipid (red arrow) released from RpfR_Ct_(PAS) coexpressed with RpfF_Bc_. The numerical values underlying panel A can be found in S1 Data. BDSF, *Burkholderia* DSF; C12:0, dodecanoic acid; LC-MS, liquid chromatography–mass spectrometry; PAS, Per-Arnt-Sim; RpfF, regulation of pathogenicity factor F; RpfR, regulation of pathogenicity factor R.
(TIF)

S4 Fig Schematic representation of the RpfR_Ct_(PAS)–BDSF interface. BDSF (sticks) is shown bound to RpfR_Ct_(PAS). Carbon atoms are colored black, oxygen atoms red, nitrogen atoms blue, and hydrogen atoms colored the same color as the atom to which they are bonded. H-bonds are depicted as dashed blue lines alongside measured distances (distances are not drawn to scale). The interaction between the BDSF and Asn202 is shown as an orange dashed line connecting the Asn202 sidechain nitrogen, with the closest BDSF carbon (C3) alongside its measured distance. The hydrophobic-binding pocket is depicted as a blue line. This molecular graphic was produced with Poseview [75]. Asn, asparagine BDSF, *Burkholderia* DSF; C3, carbon 3; PAS, Per-Arnt-Sim; RpfR, regulation of pathogenicity factor R.
(TIF)

S5 Fig Full-length wild-type RpfR_Bc_, RpfR_Bc_–S168A, RpfR_Bc_–N171A, RpfR_Bc_–R186A, and RpfR_Bc_–N201A are comparably soluble. SDS-PAGE analysis of full-length wild-type RpfR_Bc_, RpfR_Bc_–S168A, RpfR_Bc_–N171A, RpfR_Bc_–R186A, and RpfR_Bc_–N201A before and after 1 h 4 °C incubation with His_6_-Ulp1 SUMO protease. *M* protein size marker (kD). RpfR, regulation of pathogenicity factor R; SUMO, small ubiquitin-like modifier.
(TIF)

S6 Fig RpfR_Ct_(FI) is structurally distinct from PAS and GAF domains. (A) RpfR_Ct_(FI) is shown alongside the top-scoring PDBeFold and DALI server hits for PAS and GAF domains, (B) the KinA PAS-A domain (PDB: 2VLG) [76], and (C) the GAF domain of Dcsbis (PDB: 4ZMU). Structures are shown depicted as cartoon (top) and topological (bottom) models. All secondary structure elements are labeled and colored accordingly: cyan α-helix, magenta β-strand, and peach linker. Cache,Ca^2+^channels-chemotaxis receptors; DALI, Distance-matrix alignment; FI, RpfF interaction; GAF, cyclic GMP-specific phosphodiesterase-adenylyl cyclase-FhlA; PAS, Per-Arnt-Sim; PDB, Protein Data Bank; RpfR, regulation of pathogenicity factor R.
(TIF)

S7 Fig Purification of His_6_-SUMO-RpfR_Bc_(FI-PAS)-RpfF_Bc_. SDS-PAGE analysis of Ni column purification fractions. *M* protein size marker (kD), minus preinduction sample, plus postinduction sample, *P* pellet, *S* clarified lysate supernatant, *F* flow through, *W* wash, *E*_*1*_*–E*_*4*_ elutions with increasing concentrations of imidazole, *R* eluted Ni resin. Bands corresponding to His_6_-SUMO-RpfR_Bc_(FI-PAS) and RpfF_Bc_ are surrounded by red boxes. FI, RpfF interaction; PAS, Per-Arnt-Sim; RpfF, regulation of pathogenicity factor F; RpfR, regulation of pathogenicity factor R; SUMO, small ubiquitin-like modifier.
(TIF)

S8 Fig The amino acid sequence of RpfF homologues from various gram-negative species. An alignment of the amino acid sequences of RpfF from several gram-negative species that also contain an adjacent RpfR homologue are shown above a consensus sequence for identical residues (residues that are not conserved are depicted as *s). Alignment and consensus sequences were generated using CLC Sequence Viewer Version 8 (CLC bio, Aarhus, Denmark). Residues interacting with RpfR(FI) are surrounded by black boxes with residues forming a salt bridge or hydrogen-bonding interaction with RpfR(FI) highlighted blue. Secondary structure elements determined using the PyMOL algorithm [72] are shown below their corresponding sequence elements. (Orange cylinders are α-helices and green arrows are β-strands. Disordered residues are depicted as a dashed black line.) FI, RpfF interaction; RpfF, regulation of pathogenicity factor F; RpfR, regulation of pathogenicity factor R.
(TIF)

S9 Fig In the presence of RpfR_Ct_(FI), the RpfF_Bc_ substrate tunnel is devoid of fatty acid. (A) Alignment of RpfF_Bc_ from the RpfF_Bc_–RpfR_Ct_(FI) complex (gold) (RpfR_Ct_[FI] and a bound glycerol molecule are omitted for clarity) with RpfF_Bc_ alone (green) (PDB: 5FUS) [17], which contains a molecule of C12:0 present in its active site that copurified with the protein. (B) Expanded view of the area enclosed by the rectangle in A following a 20° rotation, depicting the movement of residues Phe44 and Phe88 (green and gold sticks) into space occupied by C12:0 (pink/red balls and grey sticks) in RpfF_Bc_ alone. C12:0, dodecanoic acid; FI, RpfF interaction; PDB, Protein Data Bank; Phe, phenylalanine; RpfF, regulation of pathogenicity factor F; RpfR, regulation of pathogenicity factor R.
(TIF)

S1 Table Phasing and refinement statistics. R_sym_ = Σ_h_ Σ_i_ | I_i_(h) − <I(h)>|/Σ_h_ Σ_i_ I_i_(h), in which I_i_(h) is the i^th^ measurement of h and <I(h)> is the mean for all measurements of I(h) for reflection h. R_work_ = Σ ||F_o_| − |F_c_||/Σ |F_o_| was calculated with a working set of reflections. R_free_ is R_work_ calculated using a test set of reflections. Data for the highest-resolution shells are in parentheses. Each data set was collected from a single crystal.
(DOC)

S2 Table Oligonucleotides. (DOC)

S1 Data Excel spreadsheet containing the numerical data and analysis for Figs 2G, 2H, 4B, 6A, 6B and 6C; and S1A, S1B and S3A Figs. (XLSX)

## Abbreviations

ACP: acyl carrier protein
AI: autoinducer
Arg: arginine
Asn: asparagine
BDSF: *Burkholderia* DSF
BLASTP: protein BLAST
C3: carbon 3
C12:0: dodecanoic acid
C12:0–ACP_Ec_: C12:0-charged ACP_Ec_
c-di-GMP: bis-(3′-5′)-cyclic dimeric guanosine monophosphate
Cache: Ca^2+^channels-chemotaxis receptors
CoA: Coenzyme A
DGC: diguanylate cyclase
DSF: diffusible signal factors
FI: RpfF interaction
LC-MS: Liquid chromatography–mass spectrometry
GAF: cyclic GMP-specific phosphodiesterase-adenylyl cyclase-FhlA
GMP: guanosine monophosphate
gmr: Gene modulating RNase II
holo-ACP_Ec_: *E*. *coli* 4′-phosphopantetheinyl ACP
PAS: Per-Arnt-Sim
PDB: Protein Data Bank
PDE: phosphodiesterase
PDSF: *Pseudomonas* DSF
pGpG: 5′-phosphoguanylyl-(3′,5′)-guanosine
Phe: phenylalanine
RbdA: Regulator of biofilm dispersal of Pseudomonas aeruginosa
RpfB: regulation of pathogenicity factor B
RpfF: regulation of pathogenicity factor F
RpfR: regulation of pathogenicity factor R
SAD: single-wavelength anomalous dispersion
SUMO: small ubiquitin-like modifier
