# Supplementary figures and images for: Structural basis of DSF recognition by its receptor RpfR and its regulatory interaction with the DSF synthase RpfF
Source: PLoS Biol. 2019 Feb 4;17(2):e3000123. doi: 10.1371/journal.pbio.3000123 (PMC6361424; doi:10.1371/journal.pbio.3000123)

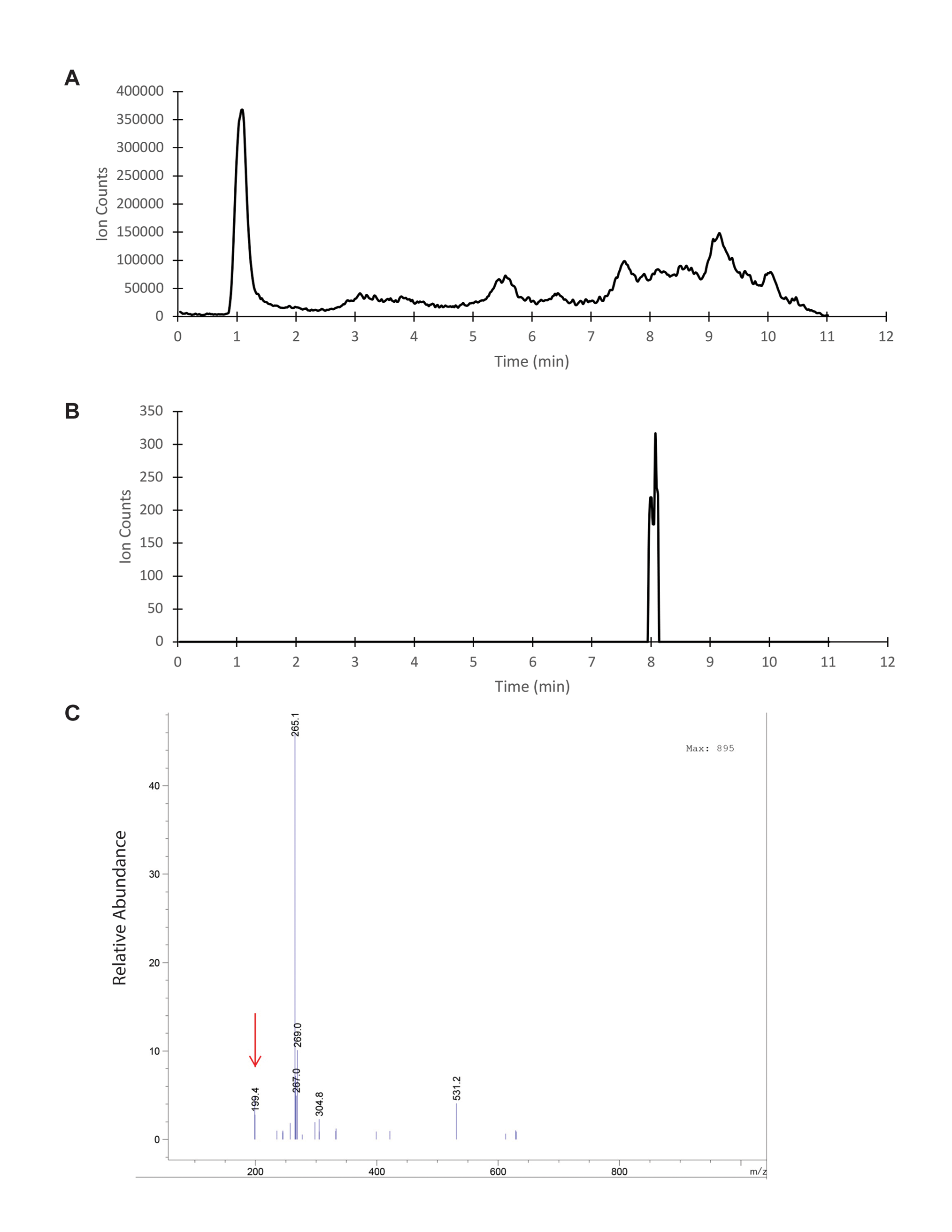

Supplement: S1 Fig — The LC-MS ionization traces of (A) total ions and (B) the extracted ion chromatogram of C12:0 in the RpfRCt(PAS) domain protein extract. (C) The mass spectrum corresponding to the C12:0 ion peak showing the negative ionization of the lipid (red arrow). The numerical values underlying panels A and B can be found in S1 Data. C12:0, dodecanoic acid; LC-MS, liquid chromatography–mass spectrometry; PAS, Per-Arnt-Sim; RpfR, regulation of pathogenicity factor R. (TIF) [file pbio.3000123.s001.tif]

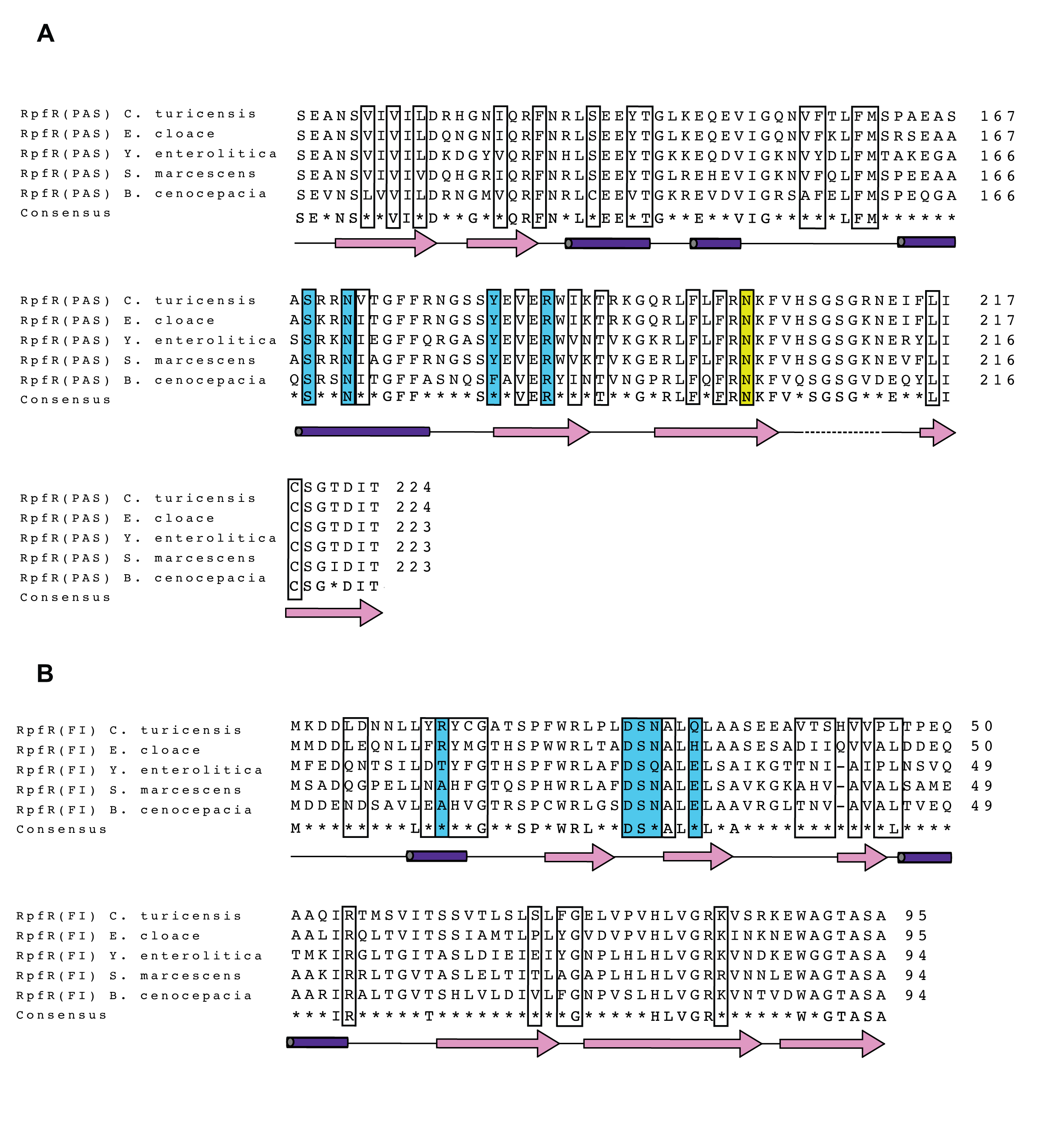

Supplement: S2 Fig — (A) An amino acid alignment and consensus sequence for conserved residues of the PAS domain from RpfR homologues was generated using CLC Sequence Viewer Version 8 (CLC bio, Aarhus, Denmark). Residues interacting with the carboxylic acid group of either C12:0 or BDSF are highlighted blue. The highly conserved Asn202 that interacts with C3 of BDSF is highlighted in yellow. The residues comprising the hydrophobic acyl–binding pocket are surrounded by unfilled black boxes. The secondary structure representation of RpfR(PAS) was determined using the PyMOL algorithm and is shown below the consensus sequence. (Purple cylinders are α-helices and pink arrows are β-strands. Disordered residues are depicted as a dashed black line.) [72] (B) An amino acid alignment and consensus sequence for conserved residues of the FI domain from RpfR homologues was generated using CLC Sequence Viewer Version 8 (CLC bio, Aarhus, Denmark). Nonconserved residues are marked with asterisks. Residues interacting with RpfF are surrounded by black boxes, with residues forming hydrogen bonds or salt bridges highlighted in blue. Interacting residues were determined by analyzing the RpfRCt(FI)–RpfFBc structure using the PISA and PDBsum servers [73,74]. The secondary structure representation of RpfR(FI) was determined using the PyMOL algorithm and is shown below the consensus sequence. (Purple cylinders are α-helices and pink arrows are β-strands.) [72] Asn, asparagine; BDSF, Burkholderia DSF; C3, carbon 3; C12:0, dodecanoic acid; FI, RpfF interaction; PAS, Per-Arnt-Sim; PDB, Protein Data Bank; PISA, Proteins, interfaces, structures, and assemblies; RpfF, regulation of pathogenicity factor F; RpfR, regulation of pathogenicity factor R. (TIF) [file pbio.3000123.s002.tif]

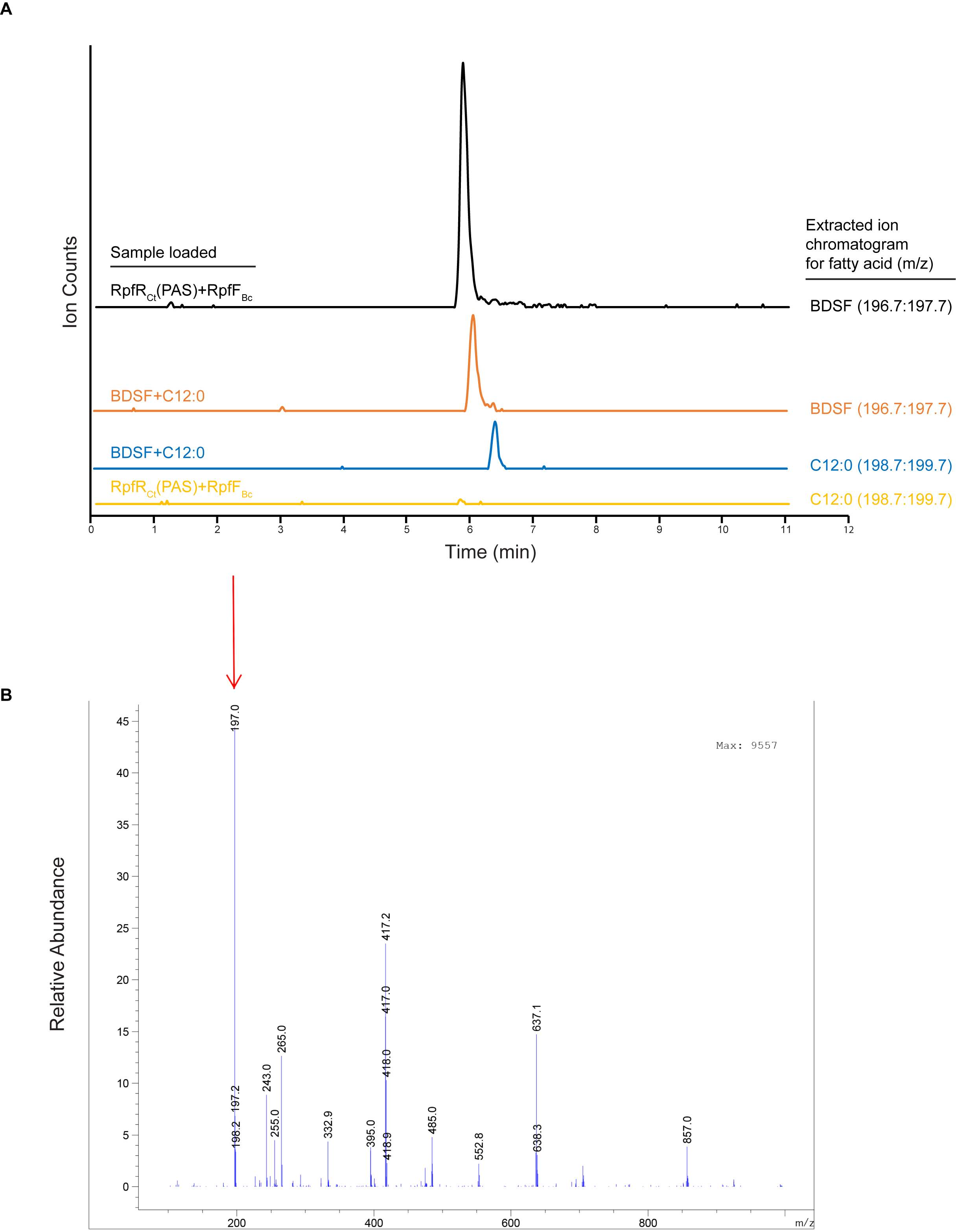

Supplement: S3 Fig — (A) Ligand released from RpfRCt(PAS) coexpressed with RpfFBc was analyzed by LC-MS, and the extracted ion chromatograms are shown for the m/z corresponding to BDSF (black curve) and C12:0 (yellow curve). For comparison, a mixture containing both BDSF (200 μM) and C12:0 (200 μM) standards was analyzed by LC-MS, and the extracted ion chromatograms are shown for the m/z corresponding to BDSF (orange curve) and C12:0 (blue curve). (B) The mass spectrum corresponding to the BDSF ion peak showing the negative ionization of the lipid (red arrow) released from RpfRCt(PAS) coexpressed with RpfFBc. The numerical values underlying panel A can be found in S1 Data. BDSF, Burkholderia DSF; C12:0, dodecanoic acid; LC-MS, liquid chromatography–mass spectrometry; PAS, Per-Arnt-Sim; RpfF, regulation of pathogenicity factor F; RpfR, regulation of pathogenicity factor R. (TIF) [file pbio.3000123.s003.tif]

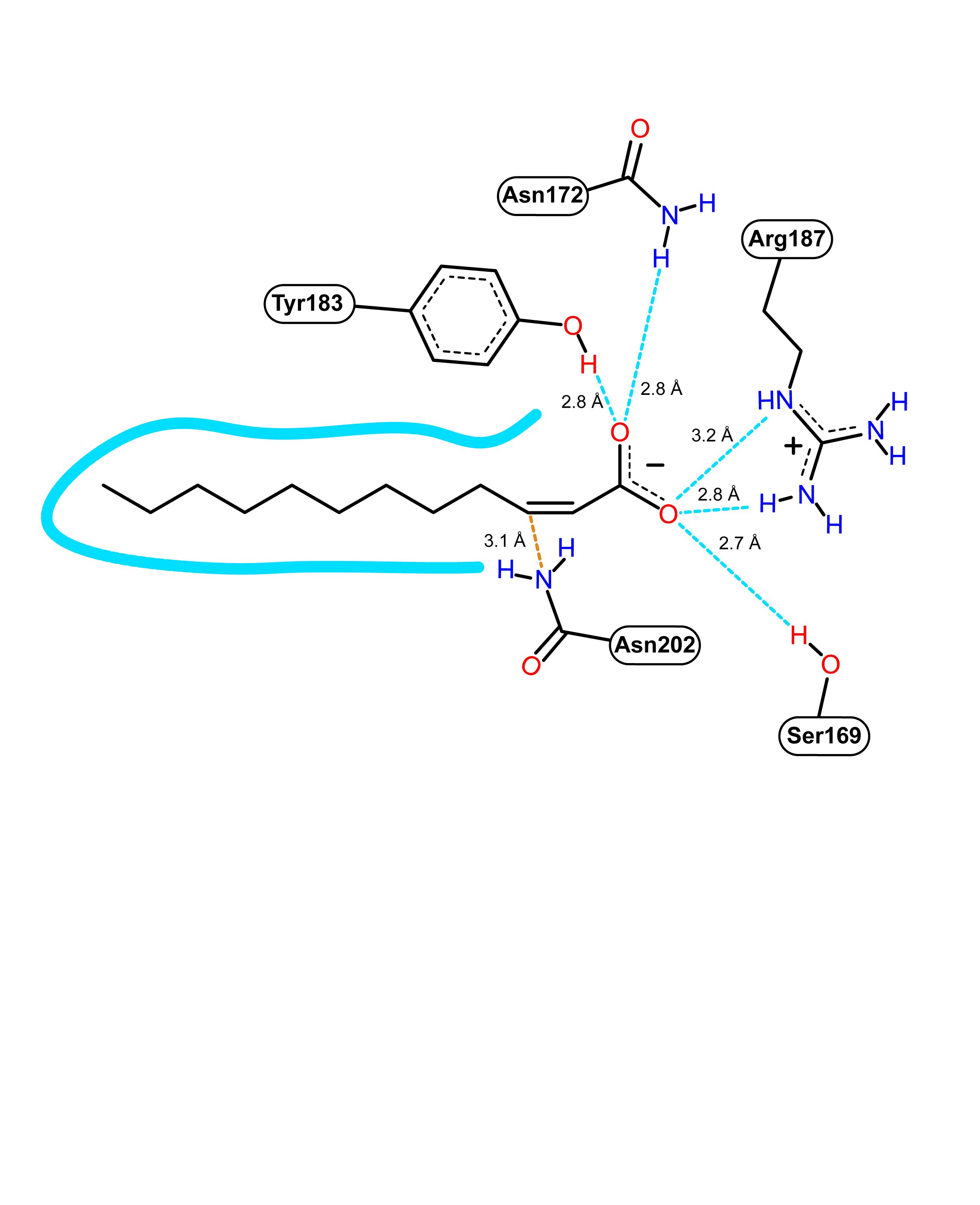

Supplement: S4 Fig — BDSF (sticks) is shown bound to RpfRCt(PAS). Carbon atoms are colored black, oxygen atoms red, nitrogen atoms blue, and hydrogen atoms colored the same color as the atom to which they are bonded. H-bonds are depicted as dashed blue lines alongside measured distances (distances are not drawn to scale). The interaction between the BDSF and Asn202 is shown as an orange dashed line connecting the Asn202 sidechain nitrogen, with the closest BDSF carbon (C3) alongside its measured distance. The hydrophobic-binding pocket is depicted as a blue line. This molecular graphic was produced with Poseview [75]. Asn, asparagine BDSF, Burkholderia DSF; C3, carbon 3; PAS, Per-Arnt-Sim; RpfR, regulation of pathogenicity factor R. (TIF) [file pbio.3000123.s004.tif]

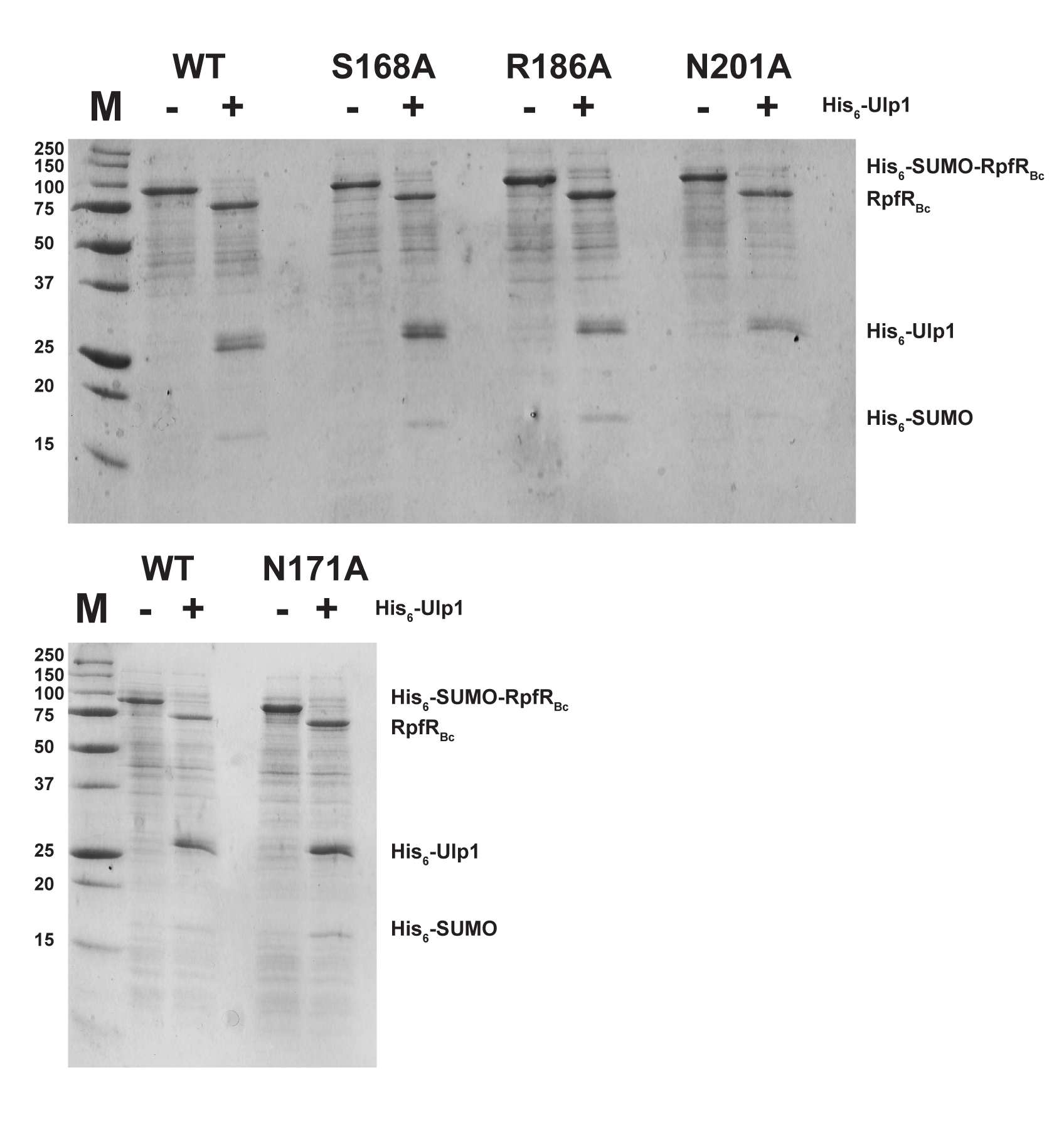

Supplement: S5 Fig — SDS-PAGE analysis of full-length wild-type RpfRBc, RpfRBc–S168A, RpfRBc–N171A, RpfRBc–R186A, and RpfRBc–N201A before and after 1 h 4 °C incubation with His6-Ulp1 SUMO protease. M protein size marker (kD). RpfR, regulation of pathogenicity factor R; SUMO, small ubiquitin-like modifier. (TIF) [file pbio.3000123.s005.tif]

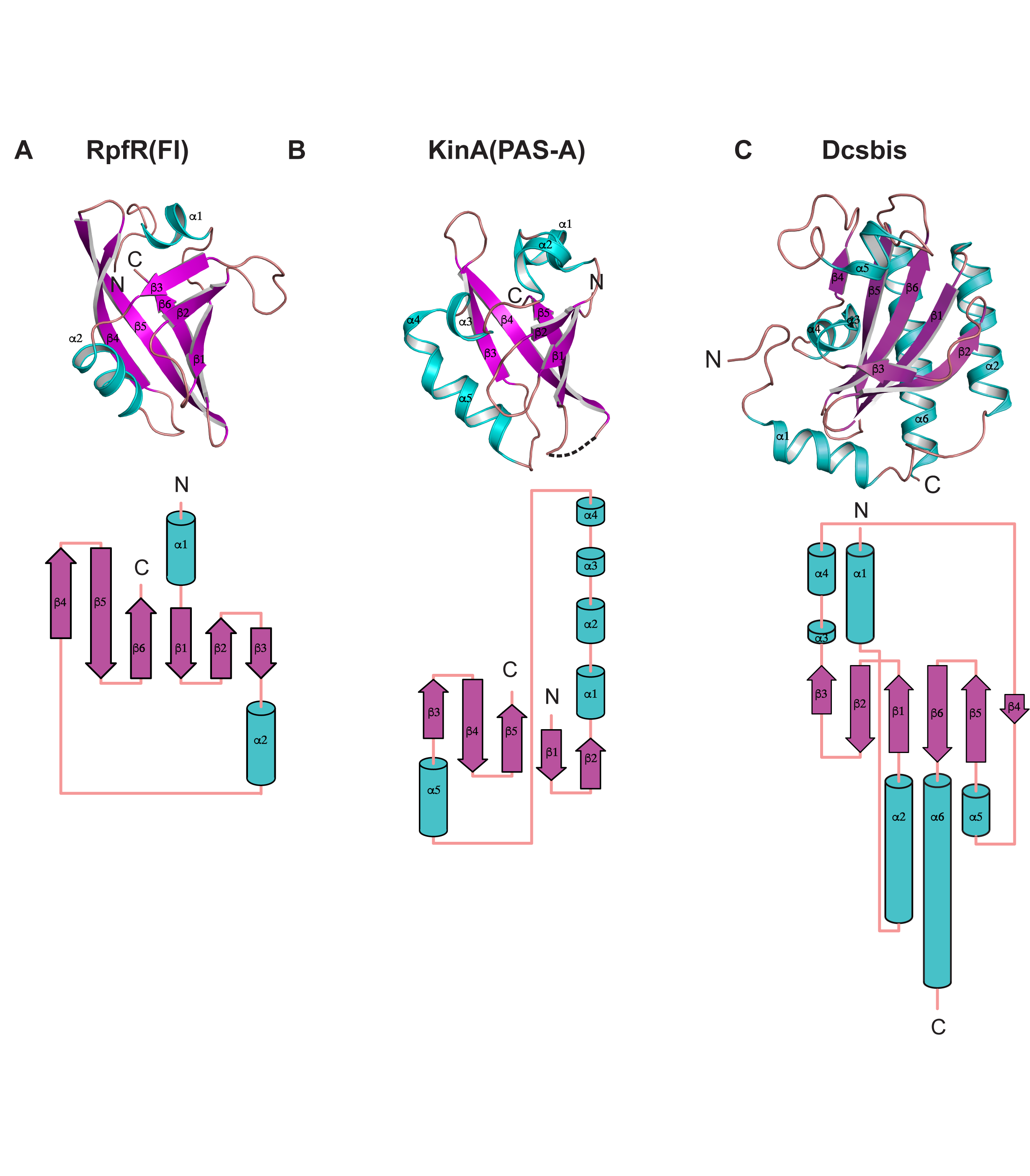

Supplement: S6 Fig — (A) RpfRCt(FI) is shown alongside the top-scoring PDBeFold and DALI server hits for PAS and GAF domains, (B) the KinA PAS-A domain (PDB: 2VLG) [76], and (C) the GAF domain of Dcsbis (PDB: 4ZMU). Structures are shown depicted as cartoon (top) and topological (bottom) models. All secondary structure elements are labeled and colored accordingly: cyan α-helix, magenta β-strand, and peach linker. Cache,Ca2+channels-chemotaxis receptors; DALI, Distance-matrix alignment; FI, RpfF interaction; GAF, cyclic GMP-specific phosphodiesterase-adenylyl cyclase-FhlA; PAS, Per-Arnt-Sim; PDB, Protein Data Bank; RpfR, regulation of pathogenicity factor R. (TIF) [file pbio.3000123.s006.tif]

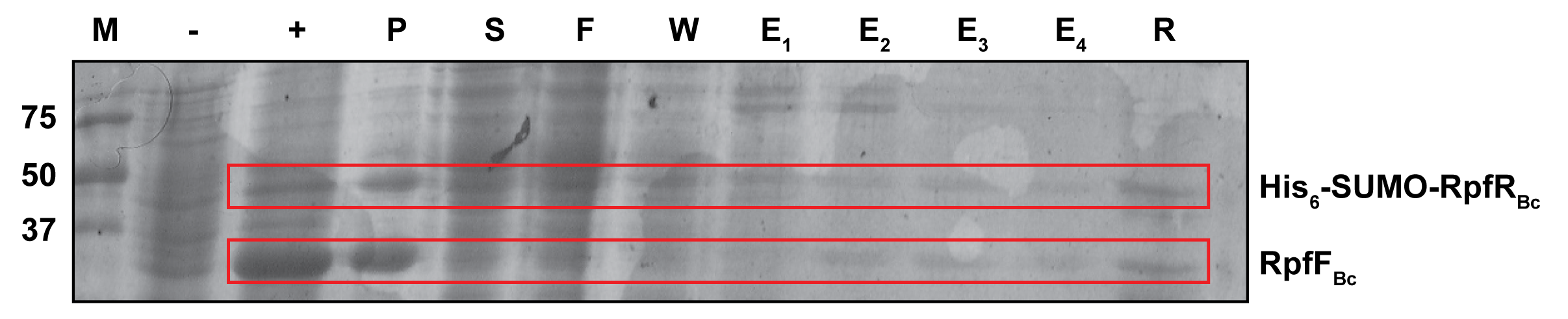

Supplement: S7 Fig — SDS-PAGE analysis of Ni column purification fractions. M protein size marker (kD), minus preinduction sample, plus postinduction sample, P pellet, S clarified lysate supernatant, F flow through, W wash, E1–E4 elutions with increasing concentrations of imidazole, R eluted Ni resin. Bands corresponding to His6-SUMO-RpfRBc(FI-PAS) and RpfFBc are surrounded by red boxes. FI, RpfF interaction; PAS, Per-Arnt-Sim; RpfF, regulation of pathogenicity factor F; RpfR, regulation of pathogenicity factor R; SUMO, small ubiquitin-like modifier. (TIF) [file pbio.3000123.s007.tif]

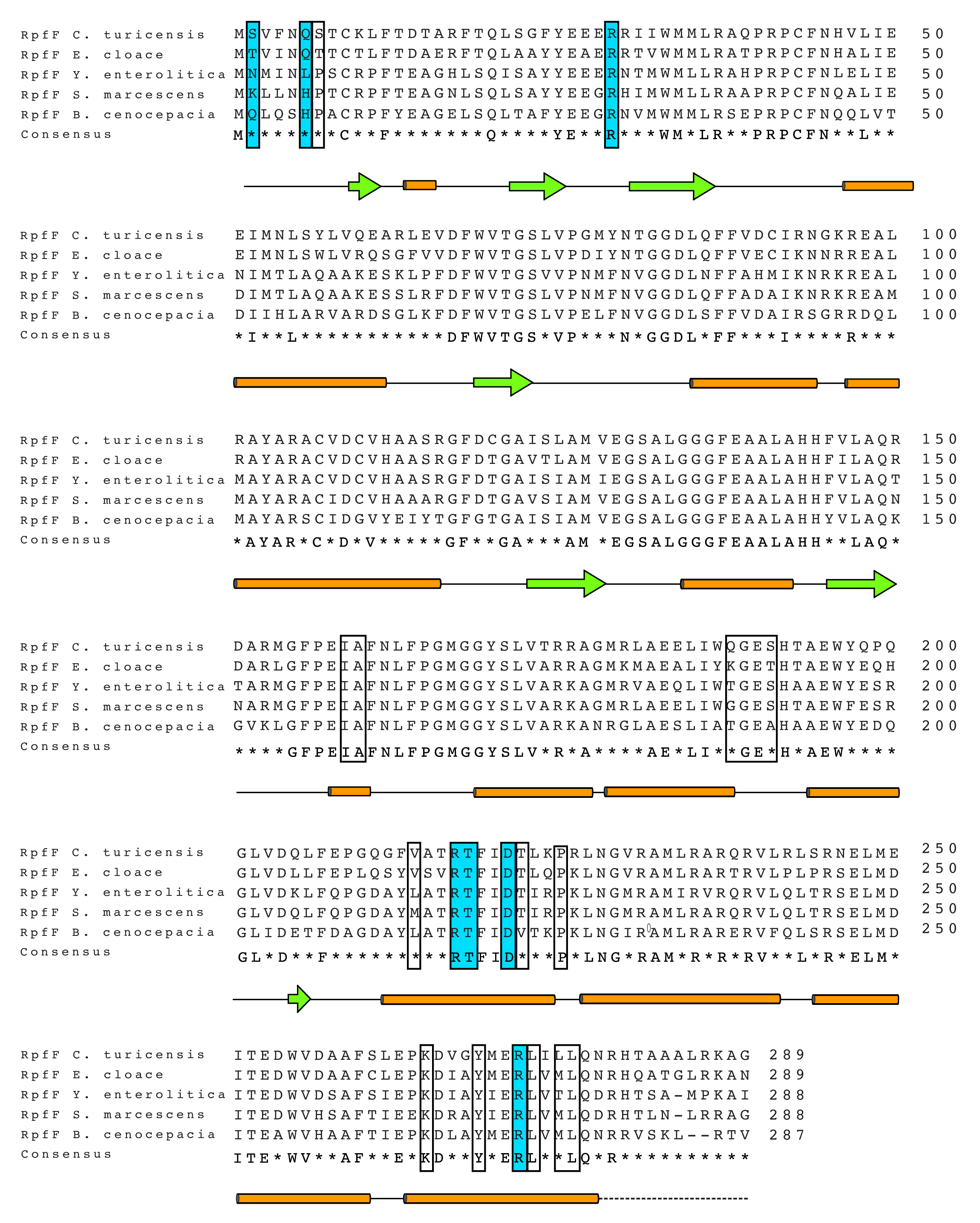

Supplement: S8 Fig — An alignment of the amino acid sequences of RpfF from several gram-negative species that also contain an adjacent RpfR homologue are shown above a consensus sequence for identical residues (residues that are not conserved are depicted as *s). Alignment and consensus sequences were generated using CLC Sequence Viewer Version 8 (CLC bio, Aarhus, Denmark). Residues interacting with RpfR(FI) are surrounded by black boxes with residues forming a salt bridge or hydrogen-bonding interaction with RpfR(FI) highlighted blue. Secondary structure elements determined using the PyMOL algorithm [72] are shown below their corresponding sequence elements. (Orange cylinders are α-helices and green arrows are β-strands. Disordered residues are depicted as a dashed black line.) FI, RpfF interaction; RpfF, regulation of pathogenicity factor F; RpfR, regulation of pathogenicity factor R. (TIF) [file pbio.3000123.s008.tif]

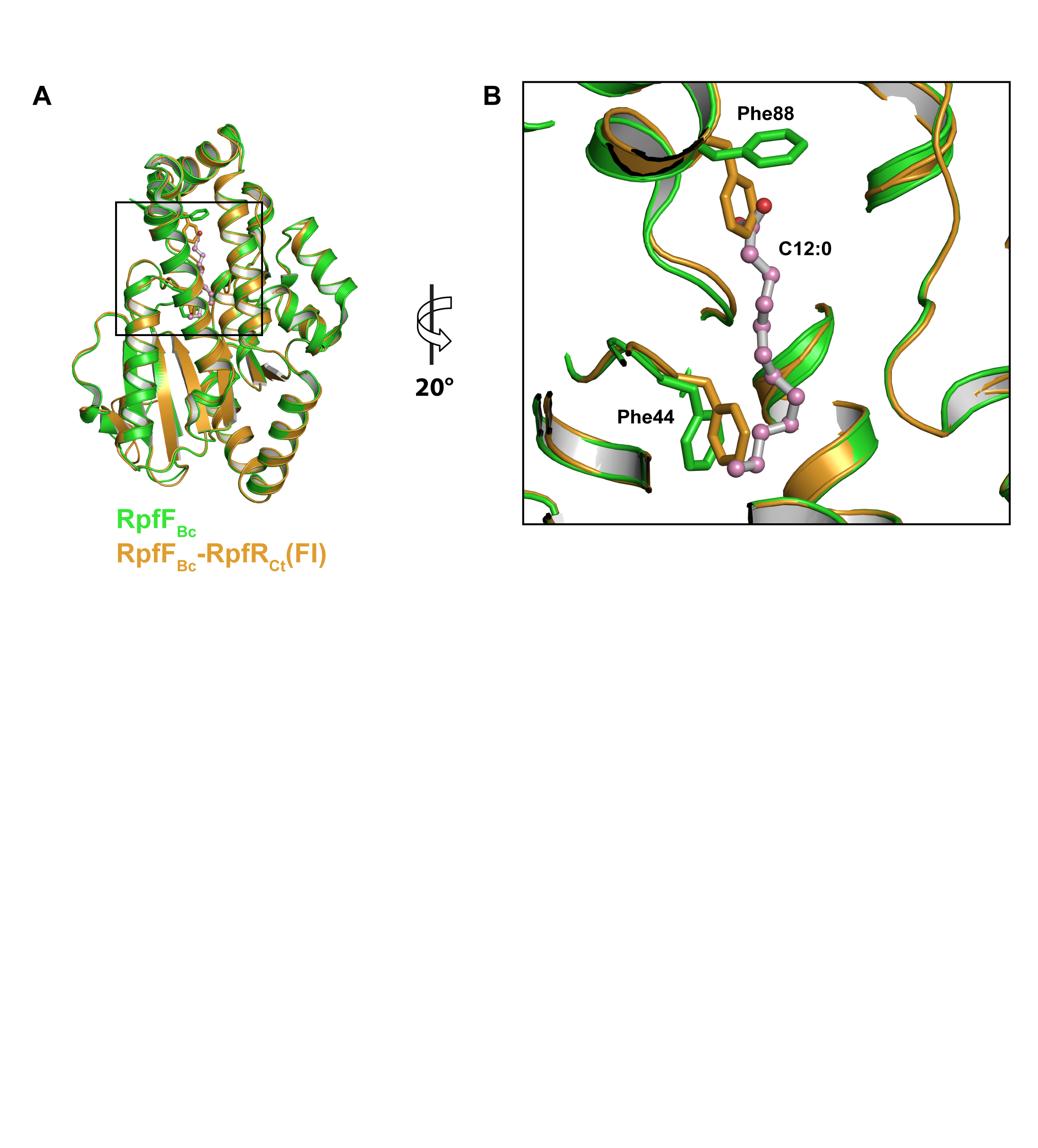

Supplement: S9 Fig — (A) Alignment of RpfFBc from the RpfFBc–RpfRCt(FI) complex (gold) (RpfRCt[FI] and a bound glycerol molecule are omitted for clarity) with RpfFBc alone (green) (PDB: 5FUS) [17], which contains a molecule of C12:0 present in its active site that copurified with the protein. (B) Expanded view of the area enclosed by the rectangle in A following a 20° rotation, depicting the movement of residues Phe44 and Phe88 (green and gold sticks) into space occupied by C12:0 (pink/red balls and grey sticks) in RpfFBc alone. C12:0, dodecanoic acid; FI, RpfF interaction; PDB, Protein Data Bank; Phe, phenylalanine; RpfF, regulation of pathogenicity factor F; RpfR, regulation of pathogenicity factor R. (TIF) [file pbio.3000123.s009.tif]
