# Supplementary material for: Structural basis of DSF recognition by its receptor RpfR and its regulatory interaction with the DSF synthase RpfF
Source: PLoS Biol. 2019 Feb 4;17(2):e3000123. doi: 10.1371/journal.pbio.3000123 (PMC6361424; doi:10.1371/journal.pbio.3000123)
Supplement: S1 Table — Rsym = Σh Σi | Ii(h) − <I(h)>|/Σh Σi Ii(h), in which Ii(h) is the ith measurement of h and <I(h)> is the mean for all measurements of I(h) for reflection h. Rwork = Σ ||Fo| − |Fc||/Σ |Fo| was calculated with a working set of reflections. Rfree is Rwork calculated using a test set of reflections. Data for the highest-resolution shells are in parentheses. Each data set was collected from a single crystal. (DOC) [file pbio.3000123.s010.doc]

**Table S1.** Data collection and refinement statistics.

|  | **SeMet SAD** | **Native** | **Native** |
| --- | --- | --- | --- |
|  | RpfR(FI)_Ct_ | RpfR(FI)_Ct_ | RpfR(FI)_Ct_-RpfF_Bc_ |
| **Data collection** |  |  |  |
| Space group | P 2_1_ 2_1_ 2 | P 2_1_ 2_1_ 2 | R 3 2: h |
| Cell dimensions |  |  |  |
| a, b, c (Å) | 55.09, 53.62, 28.19 | 54.98, 53.64, 28.20 | 144.94, 144.94, 116.91 |
| α, β, γ (°) | 90.0, 90.0, 90.0 | 90.0, 90.0, 90.0 | 90.0, 90.0, 120.0 |
| Resolution (Å) | 28.20-1.10 (1.22-1.10) | 28.20-1.19 (1.21-1.19) | 50.00-2.0 (2.03-2.00) |
| Wavelength (Å) | 0.97915 | 1.18076 | 0.97946 |
| Completeness (%) | 99.6 (99.5) | 96.7 (90.0) | 100.0 (100.0) |
| R_sym_ (%) | 9.1 (40.4) | 6.7 (48.9) | 14.9 (157.2) |
| Average I / σI | 26.96 (5.58) | 15.65 (3.33) | 17.54 (2.29) |
| Redundancy | 12.8 | 6.8 | 20.2 |
| Total reflections | 15,955,68 | 461,379 | 26,222,606 |
| Unique reflections | 34,754 | 27,494 | 31,964 |
| CC_1/2_ | 0.960 | 0.889 | 0.825 |
| **SAD Phasing** |  |  |  |
| Figure of merit | 0.554 |  |  |
|  |  |  |  |
| **Refinement** |  |  |  |
| R_work_ / R_free_ (%) |  | 16.13/19.33 | 16.78/20.12 |
|  |  |  |  |
| **Number of atoms** |  |  |  |
| All atoms |  | 1,529 | 5,881 |
| Protein |  | 1,411 | 5,673 |
| Ion |  | 0 | 61 |
| Water |  | 118 | 147 |
| **Average B-factor (Å^2^)** |  |  |  |
| All atoms |  | 24.10 | 41.06 |
| Protein Chain A |  | 21.92 | 37.03 |
| Protein Chain B |  |  | 52.42 |
| Ion |  |  | 66.19 |
| Water |  | 29.99 | 40.20 |
| **R.m.s. deviations** |  |  |  |
| Bond lengths (Å) |  | 0.006 | 0.003 |
| Bond angles (°) |  | 0.865 | 0.593 |
| **Ramachandran statistics** |  |  |  |
| Favored (%) |  | 97.85 | 96.69 |
| Allowed (%) |  | 2.15 | 3.31 |
| Outliers (%) |  | 0.00 | 0.28 |
| PDB Identifier |  | 6DGA | 6DGN |
|  |  |  |  |
|  |  |  |  |

|  | **Native** | **Native** |
| --- | --- | --- |
|  | RpfR(PAS)_Ct_-C12:0 | RpfR(PAS)_Ct_-BDSF |
| **Data collection** |  |  |
| Space group | P 1 2_1_ 1 | P 1 2_1_ 1 |
| Cell dimensions |  |  |
| a, b, c (Å) | 39.68, 29.13, 41.49, | 39.93, 29.15, 41.55 |
| α, β, γ (°) | 90.0, 115.3, 90.0 | 90.0, 115.5, 90.0 |
| Resolution (Å) | 37.52-1.50 (1.56-1.50) | 50.00-2.30 (2.34-2.30) |
| Wavelength (Å) | 0.88557 | 1.54178 |
| Completeness (%) | 97.2 (92.3) | 99.9 (100.0) |
| R_sym_ (%) | 6.5(22.1) | 20.6 (61.8) |
| Average I / σI | 9.09 (3.49) | 9.33 (3.30) |
| Redundancy | 3.2 | 3.9 |
| Total reflections | 455,656 | 35,913 |
| Unique reflections | 14,012 | 4008 |
| CC1/2 | 0.928 | 0.682 |
|  |  |  |
| **Refinement** |  |  |
| R_work_ / R_free_ (%) | 14.70/17.43 | 23.21/28.27 |
|  |  |  |
| **Number of atoms** |  |  |
| All atoms | 1,809 | 1,732 |
| Protein | 1,683 | 1,659 |
| Fatty acid | 37 | 35 |
| Ion |  |  |
| Water | 89 | 38 |
| **Average B-factor (Å^2^)** |  |  |
| All atoms | 16.41 | 27.90 |
| Protein | 15.81 | 28.02 |
| Fatty acid | 24.85 | 27.62 |
| Water | 24.34 | 23.12 |
| **R.m.s. deviations** |  |  |
| Bond lengths (Å) | 0.005 | 0.003 |
| Bond angles (°) | 0.893 | 0.522 |
| **Ramachandran statistics** |  |  |
| Favored (%) | 96.97 | 96.94 |
| Allowed (%) | 3.03 | 3.06 |
| Outliers (%) | 0.00 | 0.00 |
| PDB Identifier | 6DGG | 6DGJ |
|  |  |  |
|  |  |  |
|  |  |  |
|  |  |  |
