# Supplementary material for: Structural basis of DSF recognition by its receptor RpfR and its regulatory interaction with the DSF synthase RpfF
Source: PLoS Biol. 2019 Feb 4;17(2):e3000123. doi: 10.1371/journal.pbio.3000123 (PMC6361424; doi:10.1371/journal.pbio.3000123)
Supplement: S2 Table — (DOC) [file pbio.3000123.s011.doc]

| **Name** | Sequence |
| --- | --- |
| | Primer 1 | | --- | | 5′- GAACAGATTGGTGGTATGAAGGACGATCTGGAC -3′ |
| | Primer 2 | | --- | | 5′- TGCAGTCACCCGGGCTCGATTACGCGCTCGCGGTACCCGC -3′ |
| | Primer 3 | | --- | | 5′- AGAAGGAGATATACAATGCAACTCCAATCCCAT -3′ |
| | Primer 4 | | --- | | 5′- CTTATCTAGAGCTCGTTACACCGTGCGCAGCTT -3′ |
| | Primer 5 | | --- | | 5′- GAACAGATTGGTGGTCAAGTGGTTAGCGAGGCG -3′ |
| Primer 6 | 5′- TGCAGTCACCCGGGCTCGATTAGGTAATGTCGGTACCGCT -3′ |
| | Primer 7 | | --- | | 5′- GAACAGATTGGTGGTATGGATGACGAAAACGAT -3′ |
| | Primer 8 | | --- | | 5′- TGCAGTCACCCGGGCTCGATTAGGCGGACGCCGTGCCGGC -3′ |
| | Primer 9 | | --- | | 5′- GAACAGATTGGTGGTATGCAACTCCAATCCCAT -3′ |
| | Primer 10 | | --- | | 5′- TGCAGTCACCCGGGCTCGATTACACCGTGCGCAGCTT -3′ |
| Primer 11 | 5′- GAATCTTTATTTTCAGGGAATGAGCACTATCGAAGAACG -3′ |
| | Primer 12 | | --- | | 5′- AGCTGGGTCCTAGGCTTACGCCTGGTGGCCGTTGA -3′ |
| | Primer 13 | | --- | | 5′- GAGAAATTAACTATGGCAATATTAGGTTTAGGCACGG -3′ |
| | Primer 14 | | --- | | 5′- AGCTGGGTCCTAGGCTTAACTTTCAATAATTACCG -3′ |
| Primer 15 | 5′- TGCAGTCACCCGGGCTCGATCAGGTGATGTCGATGCCCGA -3′ |
| Primer 16 | 5′- TGCAGTCACCCGGGCTCGATCAGGCGATCAGCCTGAG -3′ |
| S168AF | 5′- GCAGGGCGCGCAGGCGCGCAGCAAC- 3′ |
| S168AR | 5′- GTTGCTGCGCGCCTGCGCGCCCTGC -3′ |
| N171AF | 5′- CGCAGTCGCGCAGCGCCATCACGGGCTTCT -3′ |
| N171AR | 5′- AGAAGCCCGTGATGGCGCTGCGCGACTGCG -3′ |
| R186AF | 5′- CGTTCGCGGTCGAGGCCTACATCAACACGG -3′ |
| R186AR | 5′- CCGTGTTGATGTAGGCCTCGACCGCGAACG -3′ |
| N201AF | 5′- CCTGTTCCAGTTCCGCGCCAAGTTCGTGCAGAGC -3′ |
| N201AR | 5′- GCTCTGCACGAACTTGGCGCGGAACTGGAACAGG -3′ |

**Table S2.** Oligonucleotides.
